# GSK-3*β*-induced Tau pathology drives hippocampal neuronal cell death in Huntington's disease: involvement of astrocyte–neuron interactions

**DOI:** 10.1038/cddis.2016.104

**Published:** 2016-04-28

**Authors:** F L'Episcopo, J Drouin-Ouellet, C Tirolo, A Pulvirenti, R Giugno, N Testa, S Caniglia, M F Serapide, G Cisbani, R A Barker, F Cicchetti, B Marchetti

**Affiliations:** 1 Neuropharmacology Section, OASI Institute for Research and Care on Mental Retardation and Brain Aging, Troina (EN), Italy; 2 Department of Biomedical and Biotechnological Sciences (BIOMETEC), Pharmacology and Physiology Sections, University of Catania, Via S. Sofia 64, Catania 95125, Italy; 3 Wallenberg Neuroscience Center, Division of Neurobiology and Lund Stem Cell Center, Lund University, BMC A11, Lund S-221 84, Sweden; 4 Department of Clinical and Experimental Medicine, Mathematics and Computer Science Section, University of Catania, Viale A. Doria, Catania 95125, Italy; 5 Centre de Recherche du CHU de Québec (CHUQ), Axe Neuroscience, Québec, QC G1V 4G2, Canada; 6 John van Geest Centre for Brain Repair, Department of Clinical Neuroscience, University of Cambridge, Cambridge CB2 0PY, UK; 7 Département de Psychiatrie and Neurosciences, Université Laval, Québec, QC G1V 0A6, Canada

## Abstract

Glycogen synthase kinase-3*β* (GSK-3*β*) has emerged as a critical factor in several pathways involved in hippocampal neuronal maintenance and function. In Huntington's disease (HD), there are early hippocampal deficits both in patients and transgenic mouse models, which prompted us to investigate whether disease-specific changes in GSK-3*β* expression may underlie these abnormalities. Thirty-three postmortem hippocampal samples from HD patients (neuropathological grades 2–4) and age- and sex-matched normal control cases were analyzed using real-time quantitative reverse transcription PCRs (qPCRs) and immunohistochemistry. *In vitro* and *in vivo* studies looking at hippocampal pathology and GSK-3*β* were also undertaken in transgenic R6/2 and wild-type mice. We identified a disease and stage-dependent upregulation of GSK-3*β* mRNA and protein levels in the HD hippocampus, with the active isoform pGSK-3*β*-Tyr^216^ being strongly expressed in dentate gyrus (DG) neurons and astrocytes at a time when phosphorylation of Tau at the AT8 epitope was also present in these same neurons. This upregulation of pGSK-3*β*-Tyr^216^ was also found in the R6/2 hippocampus *in vivo* and linked to the increased vulnerability of primary hippocampal neurons *in vitro*. In addition, the increased expression of GSK-3*β* in the astrocytes of R6/2 mice appeared to be the main driver of Tau phosphorylation and caspase3 activation-induced neuronal death, at least in part via an exacerbated production of major proinflammatory mediators. This stage-dependent overactivation of GSK-3*β* in HD-affected hippocampal neurons and astrocytes therefore points to GSK-3*β* as being a critical factor in the pathological development of this condition. As such, therapeutic targeting of this pathway may help ameliorate neuronal dysfunction in HD.

Huntington's disease (HD) is an autosomal-dominant neurodegenerative disorder of the CNS that is characterized by progressive neurological deficits, including involuntary movements (e.g., chorea, dystonia and gait abnormalities), psychiatric disturbances and cognitive decline.^1^ Notably, cognitive impairments appear early in the disease course and profoundly impact on the patients' quality of life.^2, 3^ This clinical profile reflects the fact that while the major pathology of HD involves the striatum and deep layers of the cerebral cortex,^1, 4, 5, 6, 7^ it is not restricted to this site but involves many other areas of the CNS from disease onset, including the hippocampus.^8, 9, 10^ Both neuronal loss in the hippocampal CA1 region and volumetric reduction on MRI have been reported in patients,^8, 9, 10^ which are also seen in animal models of the disease. All of this suggests that there is a selective vulnerability of hippocampal neurons to the disease process that may explain some of the cognitive and psychiatric deficits commonly seen in HD.^11^

The genetic basis of HD involves a CAG repeat expansion in exon 1 of the huntingtin (Htt) gene, leading to an abnormally long polyglutamine (polyQ) tract in Htt, a protein widely expressed throughout the brain and peripheral tissues.^4, 12^ This mutant form of the huntingtin protein (mHtt) ultimately forms aggregates in a variety of cell types with pathological consequences impacting on a wide range of cellular processes, all of which lead to neuronal dysfunction and eventually cell loss.^4, 5, 6, 7^ In astrocytes, the accumulation of mHtt alters several fundamental glial properties that are critical for neuronal survival, and thus this cell may also indirectly contribute to increase neuronal vulnerability and/or neuronal cell death.^13, 14, 15, 16, 17, 18^

Compelling evidence has been recently provided for the existence of Tau-related pathology as a contributing factor to the cognitive deficits seen in patients with HD. Postmortem analysis of HD brains has revealed an increase both in total Tau and its phosphorylated form, as well as its accumulation within mHtt-positive inclusions,^19^ including in the hippocampus. Two independent reports followed demonstrating cortical Tau hyperphosphorylation in the R6/2 transgenic (TG) mouse model of HD.^20, 21^ Furthermore, we showed that the rate of cognitive decline in a large cohort of HD patients was greater in those possessing the H2 haplotype of the Tau gene (microtubule-associated protein Tau (MAPT)) compared with those with the H1 haplotype.^22^

It is thus becoming increasingly clear that HD is a tauopathy at some level,^19, 20, 21, 22, 23^ although the molecular pathways involved in this process remain largely obscure. In this respect, it is of interest to note that activation of glycogen synthase kinase-3*β* (GSK-3*β*) has a role in the phosphorylation of Tau (MAPT), triggering cytoskeleton destabilization, Tau aggregation and neuronal dysfunction/death.^24, 25, 26, 27, 28, 29, 30, 31, 32, 33, 34^ Furthermore, upregulation of GSK-3*β* also promotes astroglial activation, astrocyte and microglia migration and increased expression of proinflammatory mediators,^35, 36, 37^ all of which may impair neuron–glial interactions leading to exacerbation of neuronal vulnerability/loss.^38, 39^ As such, this signaling molecule may have a critical role in mediating some of these Tau-related aspects of HD pathology, especially given that several recent studies have reported that GSK-3*β* signaling is dysregulated in cellular and rodent models of HD and that GSK-3 inhibitors prevent cellular polyQ toxicity caused by the HD mutation.^40, 41, 42, 43, 44, 45^ However, no clear consensus has emerged regarding whether GSK-3 activity is elevated or decreased in different HD-affected brain regions.^40, 41, 42, 43, 44, 45, 46, 47^ We thus sought to resolve this by studying one affected brain region in HD, the hippocampus, in both TG mice and human postmortem tissue, especially as very little is known about GSK-3*β* transcription as well as the kinase active form of it, phosphorylated GSK-3 beta at Tyrosine 216 (pGSK-3*β*-Tyr^216^), which critically mediates neuronal–glial dysfunction/death.^27, 31, 33, 38, 39, 48, 49, 50, 51, 52^

## Results

### Tau pathology coincides with neuronal loss in the hippocampus of HD patients

**Figure 1:**
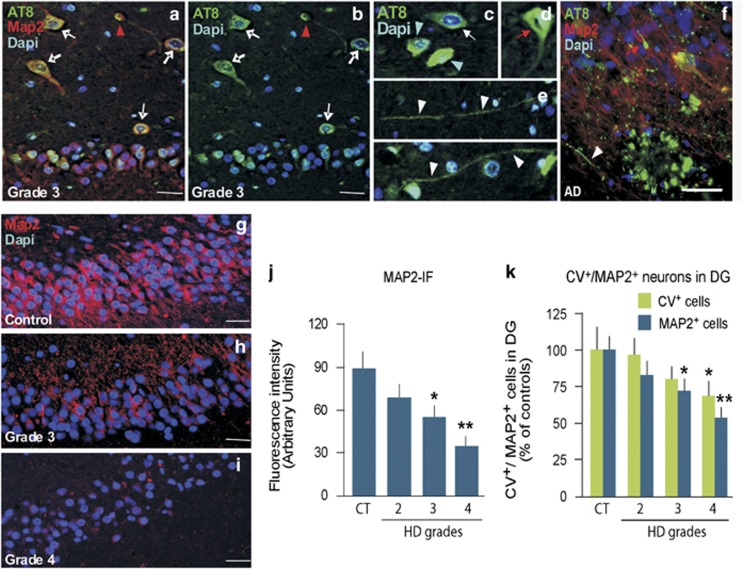
Hyperphosphorylated pathological Tau (AT8) expression coincides with the loss of MAP2-IR in the human HD hippocampus. Hippocampal slide series from HD (*n*=5 cases/severity Grade) and age- and sex-matched CT (*n*=6) were studied. Immunofluorescent staining was carried out on serial paraffin-embedded hippocampal tissue sections (6 *μ*m in thickness). Cresyl violet staining was used to count the number of neurons present in the GCL. (**a**–**e**) Representative images of triple immunofluorescent labeling (**a** and **f**) for MAP2^+^ neurons (in red) expressing phosphorylated Tau (AT8, in green) and DAPI (blue). Different morphologies of AT8^+^ cells, including ring-like perinuclear (**b**, **c**, white arrow), flame (**d**, red arrow) and globular inclusions (**b**, red arrowhead) as well as neuropil threads (**e**) can be seen in the GCL and hilus of a Grade 3 HD patient and as shown in the DG and hilus of an Alzheimer's disease (AD) patient (**f**) for comparative purposes. (**g**–**i**) MAP2 and DAPI immunofluorescence in a CT (**g**), Grade 3 (**h**) and Grade 4 (**i**) HD case with quantification (**j** and **k**) revealing a stage-dependent neuronal decrease in the DG. This result was further confirmed using a quantification of cresyl violet-stained cells against a MAP2 labeling (**k**). Statistical analysis was performed by one-way analysis of variance with Newman–Keuls Multiple Comparison *posthoc* test. Statistical differences (mean±S.D.) in panels (**j** and **k**) *P<0.05 *versus* CT; **P<0.01 *versus* CT. Scale bars in panels (**a**–**e**), (**g**–**i**)=50 *μ*m, (**f**)=25 *μ*m

In accordance with recent reports of Tau phosphorylation at the phospho-PHF-Tau (AT8) epitope in the cortex and striatum of human HD brains,^23^ we observed the presence of AT8 neuronal inclusions in the granule cell layer (GCL) and hilus of HD hippocampal sections but not of control (CT; Figures 1a–e). Different morphologies of AT8-positive (AT8^+^) cells were observed, including ring-like perinuclear, flame shaped and globular morphologies, together with numerous neuropil threads (Figures 1a–e), reminiscent of that observed in the hippocampus of Alzheimer's disease patients (Figure 1f). These AT8^+^ neuronal inclusions were accompanied by a progressive decrease in the number of cresyl violet-positive (CV^+^) and microtubule-associated protein-positive (MAP2^+^) neurons in the GCL of HD as compared with CT hippocampal sections (Figures 1g and h), suggesting that pathological Tau expression could contribute to the loss of neurons at this site in HD patients.

### Expression levels of GSK-3*β* and its kinase active form pGSK-3*β*-Tyr^216^ are both increased in the human HD hippocampus

Because GSK-3*β* can hyperphosphorylate Tau (pTau) at the majority of its sites, including AT8,^24, 25, 26, 27, 28, 29, 30, 31, 32, 33, 34^ we quantified GSK-3*β* levels in the HD and CT hippocampal samples (Table 1)^53^ using quantitative PCR (qPCR). We first analyzed the expression level and stability of 19 candidate reference genes (Supplementary Table S1) by the geNorm,^54^ NormFinder^55^ and BestKeeper programmes,^56^ which ranked RPL13A and RPLP0 as the most stable reference genes (Supplementary Figure S1 and Supplementary Table S2). We next validated the normalization accuracy of RPLP0 using the quantification of glial fibrillary acidic protein (GFAP) gene expression^1, 6, 16^ and supported expression which showed a significant increase both at the mRNA and protein levels in HD as compared with CT hippocampal samples (Supplementary Figure S3).

**Figure 2:**
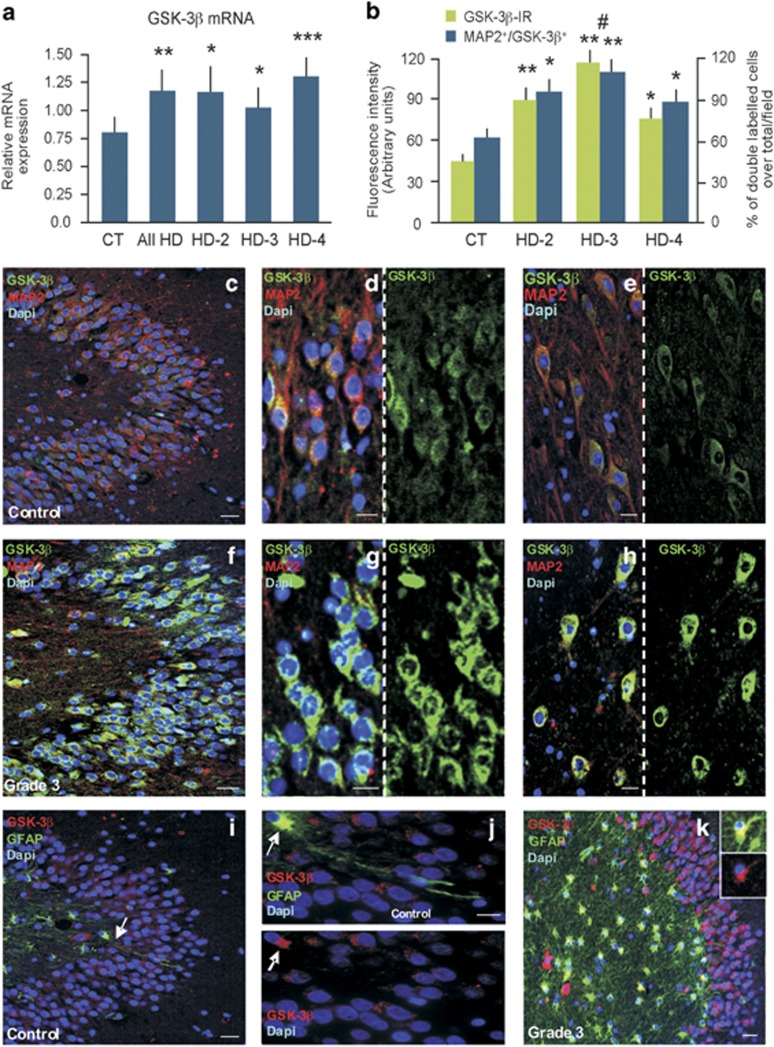
Upregulation of GSK-3*β* mRNA and protein levels in GCL neurons and astrocytes in the human HD hippocampus. (**a** and **b**) qPCR analysis of GSK-3*β* transcription and immunoreactivity in CT and HD. The characteristics of the human hippocampal samples are described in Table 1. Statistical analyses were carried out between the total CT and HD groups (*n*=32) and across grade severity compared with the CT group. Statistical differences in mRNA expression levels (mean±S.D.) were determined using a one-way analysis of variance followed by correction for multiple testing using the Tukey's Multiple Comparison test; *P<0.05, **P<0.001; ***P=0.0001. (**b**) Quantification of GSK-3*β* FI and the percentage of MAP2^+^/GSK-*β*^+^ cells over the total number of MAP2^+^ neurons/field (means±S.D.), showing a significant increase of GSK-3*β*-IR and of double labeled MAP2^+^/GSK-3*β*^+^ neurons, which was the highest in Grade 3 brains. *P<0.05 and **P<0.01 *versus* CT; #*P*<0.05 *versus* Grade 4. (**c**–**h**) Representative images of triple labeling with MAP2 (red), GSK-3*β* (green) and DAPI (blue) depicting GSK-3*β* expression in the MAP2^+^ neurons in the granular and pyramidal cell layer both in a CT (**c**–**e**) and a Grade 3 HD brain (**f**–**h**). (**i**–**k**) Triple labeling showing GSK-3*β* expression (red) in GFAP^+^ astrocytes (green) and DAPI (blue) in hippocampal sections from CT and HD (**i**–**k**). White arrows (**i**, **j**) show a GSK-3*β* (red) expressing GFAP+ astrocyte (green) in the GCL of a representative control hippocampal section. Scale bars in panels (**c**), (**f**), (**i**), (**k**)=50 *μ*m; (**d**), (**e**), (**g**), (**h**), (**j**)=25 *μ*m

Using RPLP0 as a reference gene (Figure 2a), we then found that GSK-3*β* mRNA transcripts were significantly increased in the whole HD group (F=14.25; *P*=0.00063), as well as in the separate HD Grades 2–4 relative to the CT cases, with the Grade 4 group reaching the highest level of significance (F=24.385; *P*<0.001) as compared with Grade 2 (F=5.775; *P*=0.036) and Grade 3 (F=6.036; *P*=0.027) (Figure 2a).

We next investigated whether GSK-3*β* overexpression observed in HD hippocampal samples was associated with an increased expression of GSK-3*β* at the protein level (Figures 2b–k). In CT hippocampi, GSK-3*β* co-localized primarily with MAP2^+^ neurons in the GCL (Figures 2c and d) and in pyramidal cells of Ammon's horn (Figure 2e). MAP2, a neuron-specific phosphoprotein and a substrate for GSK-3*β*,^28^ was expressed at higher levels within the cell bodies and dendrites in the CT brains, with the GSK-3*β*-IR being preferentially distributed in the MAP2^+^ neuronal cytoplasm with little staining in the nucleus (Figures 2c–e). In HD cases, there was a significant increase of GSK-3*β*-IR and of double-labeled MAP2^+^/GSK-3*β*^+^ neurons, which was highest in Grade 3 brains (*P*<0.01, Figure 2b). As observed, a strong GSK-3*β*-IR signal was detected in the MAP2^+^ neuronal cytoplasm of GCL neurons (Figures 2f and g) and pyramidal cells of Ammon's horn (Figure 2h). MAP2 expression was markedly reduced within the cell bodies and dendrites of HD (Figures 2g and h) as compared with CT neurons (Figures 2d and e). Double labeling with GFAP and GSK-3*β* revealed GSK-3*β* expression in a restricted number of astrocytes in CT (Figures 2i and j), whereas its expression was detected in a much greater number of GFAP+ astrocytes in HD (Figure 2k), showing a significantly increased (*P*<0.01) GFAP-IR as well as cell density (*P*<0.01) in the dentate gyrus (DG) as compared with CT (Supplementary Figures S3B–D).

**Figure 3:**
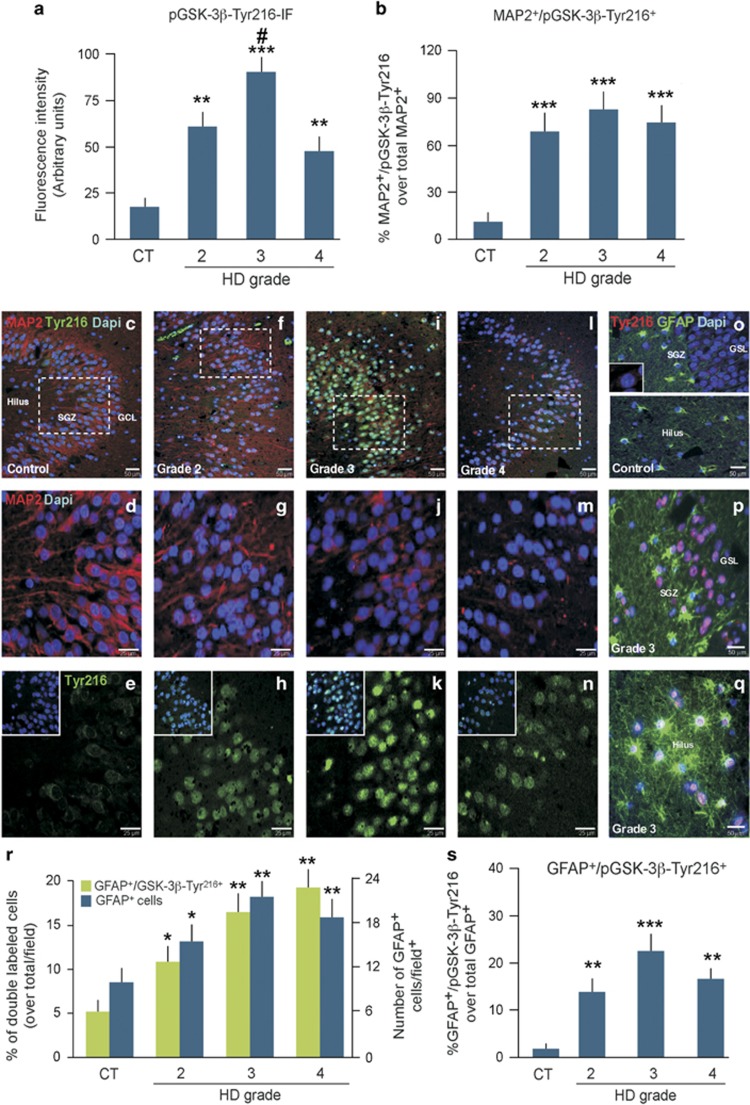
Increased expression and translocation of the GSK-3*β*-Tyr^216^ isoform in human HD hippocampus. (**a**) pGSK-3*β*-Tyr^216^ immunofluorescence intensity and (**b**) the number of MAP2^+^ cells expressing pGSK-3*β*-Tyr^216^ in HD *versus* CT (*n*=16). Statistical differences were determined by a one-way analysis of variance (ANOVA) with Newman–Keuls Multiple Comparison *posthoc* test. **P<0.01 *versus* CT; ***P<0.001 *versus* CT; #*P*<0.05 *versus* Grade 4. Note the sharp increase of MAP2^+^ cells expressing pGSK-3*β*-Tyr^216^ in Grades 2–4 HD *versus* CT (**b**), with the highest level of pGSK-3*β*-Tyr^216^-IR being detected in Grade 3 HD (**a**). (**c**–**n**) Representative images depicting triple immunofluorescent labeling of neurons for MAP2 (red), pGSK-3*β*-Tyr^216^ (green) and DAPI (blue) in a (**c**–**e**) CT, (**f**–**h**) Grade 2, (**i**–**k**) Grade 3 and (**l**−**n**) Grade 4 HD brain. p-GSK-3*β*Tyr^216^ expression in MAP2^+^ neurons have a preferential nuclear distribution in HD cases (**f**–**n**), whereas in CT faint immunoreactivity is located within the cytoplasm (**c**–**e**). (**o**–**q**) Triple immunofluorescent staining of pGSK-3*β*-Tyr^216^ (in red), GFAP (in green) and DAPI (blue) revealed scarce cytoplasmic or nuclear expression of the active kinase in the majority of astrocytes in the DG and hilus of CT subjects (**o**). In HD, both pGSK-3*β*-Tyr^216^-negative and pGSK-3*β*-Tyr^216^-positive astrocytes were observed in the DG and hilus (**p** and **q**). (**r** and **s**) The number of astrocytes was greater in the HD hippocampi as compared with CT as well as the level of pGSK-3*β*-Tyr^216^ expression. Statistical differences were determined by a one-way ANOVA with Newman–Keuls Multiple Comparison *posthoc* test. *P<0.05 *versus* CT; **P<0.01 *versus* CT; ***P<0.001 *versus* CT. Changes in the percentage of double-labeled GFAP+/GSK-3*β*^+^ cells over the total number of GFAP+ cells/field in DG areas (means±S.D.) showed that only a limited number of GFAP+ cells expressed GSK-3*β* in CT brains, whereas there was a significant increase (**P<0.01 *versus* CT) of double-labeled astrocytes in HD (**o**–**q**). SGZ, subgranular zone

The regulation of GSK-3*β* activity is brought about by complex mechanisms, including phosphorylation.^29, 30, 32, 33, 34, 48, 49, 50, 51, 52^ Here we focused our analysis on pGSK-3*β*-Tyr^216^ as there is substantial evidence that Tyr^216^ phosphorylation of GSK3*β* represents an important mechanism by which cellular insults can lead to neuronal death^27, 31, 33, 38, 39, 48, 49, 50^ and is known to be stably expressed in the postmortem brains.^57^ We found that, in the CT hippocampus, faint pGSK-3*β*-Tyr^216^ labeling could be observed mostly in the cytoplasm of MAP2^+^ neurons (Figures 3c–e) while in HD there was a significant increase (*P*<0.01; *P*<0.001) of the active kinase in MAP2^+^ neurons of the DG, with the highest levels detected in Grade 3 HD (Figures 3a, b and f–n). Furthermore, the distribution of pGSK-3*β*-Tyr^216^ labeling in MAP2^+^ neurons was different in HD compared with CT, with the pGSK-3*β*-Tyr^216^ staining being primarily nuclear (Figures 3h, k and n). This was accompanied by a decrease in the average MAP2 fluorescence intensity (FI) in GCL neurons at the level of the cell bodies and dendrites in the HD samples (Figures 1g, j, 2g, h, 3d, g, j and m). Double staining of pGSK-3*β*-Tyr^216^ with astroglial cell markers indicated very little cytoplasmic/nuclear expression of the active kinase in the DG and hilus of the CT hippocampus (Figures 3o, r and s), whereas pGSK-3*β*-Tyr^216^-IR signal was detected in greater numbers of HD hippocampal astrocytes (Figures 3p–s). Again, the distribution of pGSK-3*β*-Tyr^216^ labeling in HD astrocytes was primarily nuclear (Figures 3p and q). Taken together, these results show that GSK-3*β* gene transcription is upregulated in the HD hippocampus and that, at the protein level, GSK-3*β* and the active pGSK-3*β*-Tyr^216^ isoform are also increased both in neurons and astrocytes in a stage-dependent manner in the HD GCL.

### Active pGSK-3*β*-Tyr^216^ increases neuronal vulnerability to oxidative stress in R6/2 TG mouse hippocampal neurons

**Figure 4:**
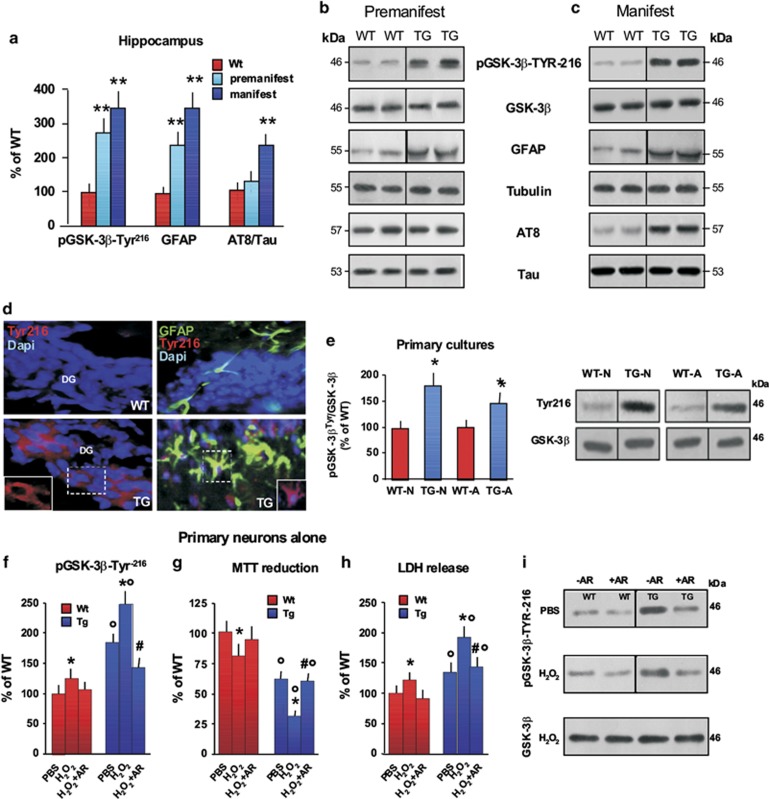
Contribution of active pGSK-3*β*-Tyr^216^ to mHtt-induced neuronal death in R6/2 hippocampus. (**a**–**c**) Hippocampi were analyzed from WT and TG mice at 3 weeks (premanifest, *n*=6 each for TG and WT) and at ≥8 weeks (manifest, *n*=6 each for TG and WT). Quantification of phosphoepitopes was performed *versus* total GSK-3*β* or total Tau, respectively, and normalized to *β*-tubulin. GFAP quantification was performed *versus β*-tubulin (as loading control). Results are expressed as the percentage of WT mice. Data are mean±S.D. Statistical differences were analyzed by analysis of variance followed by a Newman–Keuls test. **P<0.01 *versus* WT. (**b** and **c**) Two continuous lanes (side by side) from the same blot are displayed for each condition. Black borders correspond to where the images were cropped. (**d**) Representative immunofluorescent staining of hippocampal sections from 12-week-old WT and R6/2 mice showing increased pGSK-3*β*-Tyr^216^-IR in the DG of HD mice, whereas only a faint staining is observed in the WT counterparts. Triple immunofluorescent staining with DAPI (blue), GFAP (green) and pGSK-3*β*-Tyr^216^ (red) shows increased staining of the active kinase, which co-localizes with TG but not WT astrocytes. (**e**) WT and TG mice brains showing increased pGSK-3*β*-Tyr^216^ in TG as compared with WT cells and western blotting analysis of pGSK-3*β*-Tyr^216^ in primary hippocampal neurons and astrocytes showing increased pGSK-3*β*-Tyr^216^ in TG as compared with WT cells. Two representative lanes for neuronal (WT-N, TG-N) and astrocyte (WT-A, TG-A) cells are displayed. *P<0.05 *versus* WT. (**f** and **i**) Western blotting analyses of pGSK-3*β*-Tyr^216^ levels in hippocampal neurons at 7–10 DIV after a mild oxidative stress challenge (H_2_O_2_, 0.5 *μ*M) in WT and TG cultures, with or without pretreatment (−1 h) with the specific GSK-3*β* antagonist AR (5 *μ*M).^70, 73^ Two representative continuous lanes are displayed (−AR and +AR) for WT and TG cells, both in PBS- and H_2_O_2_-treated conditions. *P<0.05 *versus* PBS in WT and TG cultures, respectively; ^○^*P*<0.01 *versus* WT cultures; ^#^*P*<0.05 *versus* H_2_O_2_. (**g** and **h**): MTT reduction assay and LDH release under basal conditions (PBS) and after a mild oxidative stress challenge (H_2_O_2_, 0.5 *μ*M) in WT and TG cultures, in the absence or not of (−1 h) AR (5 *μ*M). Values are expressed relative to untreated WT cells (percentage of WT) and represent the mean±S.D. of four replicates. *P<0.05 *versus* PBS in WT and TG cultures, respectively; ^○^*P*<0.01 *versus* WT cultures; ^#^*P*<0.05 *versus* H_2_O_2_. A, astrocytes; N, neurons

In order to expand on these findings, and given the difficulty of studying causal links in disease pathways in human postmortem tissue, we next turned to the well-characterized R6/2 mouse model of HD.^58^ Using western blotting, we first quantified the levels of active pGSK-3*β*-Tyr^216^ in hippocampal tissue from wild-type (WT) and TG mice before (2.5–3 weeks) and after the onset of HD-like features (>8 weeks). We found a 3–4-fold increase of active pGSK-3*β*-Tyr^216^ in premanifest and manifest mice (Figures 4a–c) as has recently been reported in the R6/1 mouse model.^47^ In addition, GFAP and AT8 protein levels were significantly increased in R6/2 mice as compared with WT counterparts (Figures 4a–c). Consistently, astrocytic pGSK-3*β*-Tyr^216^-IR was increased in the DG of TG, as compared with that observed in WT mice (Figure 4d). This pGSK-3*β*-Tyr^216^ increase was also seen in primary hippocampal neuronal and astrocyte cell cultures derived from R6/2 mice, when compared with active kinase levels detected in WT counterparts (Figure 4e). In agreement with a previous study,^21^ phosphorylation of Tau at the AT8 epitope was not changed in premanifest mice (Figures 4a–c), whereas a significant increase was observed after the onset of the disease when compared with WT mice, suggesting that mHtt can induce Tau hyperphosphorylation with the progression of HD hippocampal pathology.

Accordingly, we next studied the impact of pGSK-3*β*-Tyr^216^ on neuronal viability in hippocampal neuronal cultures derived from WT and TG mouse brains. Notably, unlike astrocytes, neurons are highly vulnerable to mHtt *in vitro*^13, 14, 15, 16, 17, 18, 40^ (Supplementary Figure S4). Consistently, increased pGSK-*β*-Tyr^216^ protein levels (Figures 4f and i) were associated with a reduction in TG neuronal (TG-N) viability, as revealed by decreased mitochondrial activity (Figure 4g), and increased cell death (Figure 4h), as compared with TG astrocytes (TG-A), which showed no change in cell viability as compared with their WT counterparts (Supplementary Figure S4).

Because oxidative stress and mitochondrial dysfunction are critically involved in HD,^7, 59^ and given that GSK-3*β* becomes activated when apoptotic cell death is initiated,^27, 38, 39, 48, 49, 50^ we addressed the contribution of GSK-3*β* hyperactivation to the response of TG-N exposed to oxidative stress. A low dose of hydrogen peroxide (H_2_O_2_) induced only a small increase in pGSK-3*β*-Tyr^216^ in WT-N (Figures 4f and i) and a nearly 25% decrease in neuronal viability, as measured using 3-(4,5-dimethylthiazolyl-2)-2,5-diphenyltetrazolium bromide (MTT) reduction and lactate dehydrogenase (LDH) release assays (Figures 4g and h). In contrast, exposure to the same low oxidative challenge in TG neurons further increased the upregulation in GSK-3*β*-Tyr^216^ protein levels (Figures 4f and i). This was associated with an additional significant decrease of TG-N (but not TG-A) viability as indicated by the sharp decrease in mitochondrial activity and significant increase in cell death markers (Figures 4g and h,Supplementary Figure S4). Finally, pretreatment with the specific GSK-3*β* antagonist AR-AO14418 (*N*-(4-methoxybenzyl)-*N*'-(5-nitro-1,3-thiazol-2-yl)urea), AR) rescued the TG-N neurons (Figures 4f–i).

### TG astrocytes overexpressing pGSK-3*β*-Tyr^216^ lose their neurotrophic and neuroprotective properties, exacerbating neuronal loss

**Figure 5:**
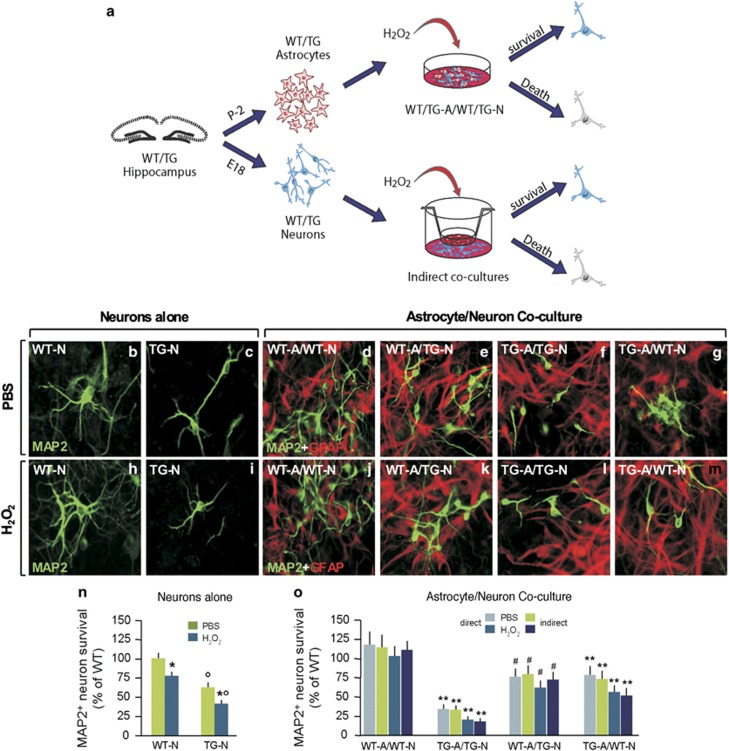
Impaired glial neurotrophic and neuroprotective properties by hyperactive pGSK-3*β*-Tyr^216^ in R6/2 astrocytes. (**a**) Schematic drawing showing the co-culture experiments. In the direct co-culture paradigm, WT/TG neurons were layered on top of WT/TG astrocytes for 7–10 DIV, allowing contact between neurons and astrocytes. In the indirect co-culture, glial inserts were added on top of the purified neurons, allowing only factor diffusion. (**b**, **c**, **h**, **i**) Representative images of MAP2+ staining in WT-N and TG-N cultured alone or in the presence (or not) of mild oxidative stress (H_2_O_2_, 0.5 *μ*M). (**d**–**g**), (**j**–**m**) Double immunofluorescent staining with GFAP and MAP2 in the direct co-culture paradigms in the absence (**d**–**g**) or presence (**j**–**m**) of a mild oxidative stress (H_2_O_2_, 0.5 *μ*M). (**n** and **o**) Neuronal survival as determined by MAP2^+^ cell counting in the different conditions. Values are expressed as the percentage of WT and represent the mean±S.D. of four replicate counts in each condition. Statistical differences were analyzed by analysis of variance followed by a Newman–Keuls test *P<0.05 *versus* PBS in WT and TG cultures, respectively; ^○^*P*<0.01 *versus* WT cultures; **P<0.01 *versus* WT-A/WT-N; ^#^*P*<0.01 *versus* TG-A/TG-N. A, astrocytes; E18, embryonic day 18; N, neurons; P-2, postnatal day 2

Because GSK-3*β* upregulation may impair astrocyte–neuron interactions,^38, 39^ we next addressed the effects of overexpressing GSK-3*β* using different astrocyte–neuron co-culture paradigms and treatments (Figure 5a). In both direct and indirect WT-N/WT-A co-cultures, we confirmed the glial neurotrophic effects, as revealed by increased MAP2 density and neurite length and branching when compared with WT-N cultured without glia (Figures 5b, d, n and o). In striking contrast to WT-A, in the TG-N/TG-A co-culture paradigms, we observed a sharp reduction of MAP2^+^ neurons, associated with reduced neurite length and branching (Figures 5e and o) as compared with WT-N/WT-A counterparts (Figures 5f and o), supporting the concept that mHtt significantly impairs the neurotrophic properties of astrocytes. Consistently, when TG-A monolayers were placed on top of WT-N, MAP2^+^ neuronal survival was reduced as compared with WT-A/WT-N co-cultures (Figures 5g and o), and conversely, when WT-A were layered on top of TG-N, we observed a significant increase in MAP2^+^ neuronal survival (Figures 5e and o). This implies that mHtt may induce specific astrocyte-derived factors/mechanisms, which are at least in part responsible for reduced neuronal survival. Remarkably, while exposure of WT-N/WT-A cultures to low doses of H_2_O_2_ was without significant effects on MAP2^+^ cell density (Figures 5j and o), a low oxidative H_2_O_2_ challenge in TG-N/TG-A co-cultures reduced the numbers of MAP2^+^ neurons (Figures 5l and o), an effect that was even more robust when compared with TG-N cultures alone (Figures 5i, n and o), thereby supporting the contention that, besides other neuronal effects, additional factors linked to the TG-A exacerbate hippocampal TG-N vulnerability.

### Depletion of neuronal GSK-3*β* attenuates Tau phosphorylation and caspase3 activation in R6/2 neurons

**Figure 6:**
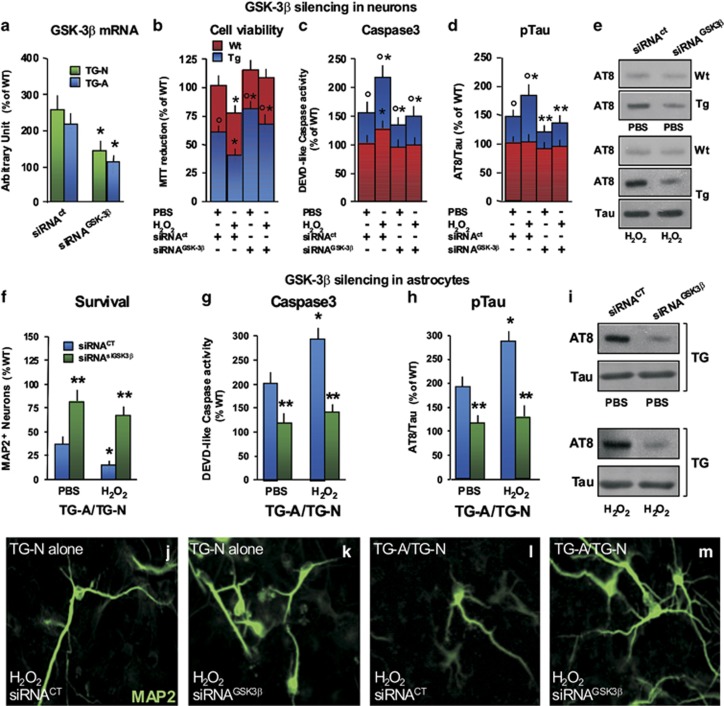
Synergistic effect of neuronal and astrocyte hyperactive GSK-3*β* in the exacerbation of Tau phosphorylation and neuronal death in R6/2 hippocampal cultures. (**a**) qPCR quantification of GSK-3*β* mRNA following a treatment with siRNA^GSK-3*β*^ or a siRNA^CT^ in neurons. *P*<0.05 *versus* siRNA^CT^ within TG-N and TG-A, respectively. (**b**) Cell viability in the presence of a mild oxidative stress, siRNA^GSK-3*β*^, siRNA^CT^ or PBS was measured with the MTT reduction assay. (**c**) Caspase3 activity was also measured using the fluorogenic substrate DEVD-AFC. (**d** and **e**) Tau phosphorylation at the AT8 epitope was determined by western blotting and quantifications performed against total Tau. Two representative continuous lanes are displayed (siRNA^CT^ and siRNA^GSK-3*β*^) for WT and TG, both in PBS- and H_2_O_2_-treated conditions. Values are expressed as a percentage of WT cells and represent the mean±S.D. of four replicate counts in each condition. Statistical differences were analyzed by analysis of variance followed by a Newman–Keuls test. *P<0.05 *versus* siRNA^CT^-PBS-treated cells, within WT and TG cultures, respectively; ^○^*P*<0.01 *versus* WT cultures; *P<0.05 *versus* siRNA^CT^-H_2_O_2_-treated cells. (**f**–**i**) Quantification of neuronal survival (**f**), caspase3 activity (**g**) as well as Tau phosphorylation at the AT8 epitope (**h**) following GSK-3*β* silencing in TG astrocytes using the indirect co-culture (TG-A/TG-N) paradigm with or without mild oxidative stress. Values are expressed as a percentage of WT cells and represent the mean±S.D. of three replicate counts in each condition. *P<0.05 *versus* PBS-treated cells; **P<0.01 *versus* siRNA^CT^ cultures. (**j**–**m**) Representative images of MAP2^+^ fluorescent staining of TG-N neurons in the presence of an H_2_O_2_ challenge when cultured alone (**j** and **k**) or with TG-A (**l** and **m**)

GSK-3*β* transgene expression induces Tau hyperphophorylation with the formation of pretangle structures in the hippocampus, as well as neuronal death, gliosis and learning deficits.^25^ We thus addressed the specific role of neuronal *versus* astrocyte GSK-3*β* by investigating the effects of a small interfering (siRNA) against GSK-3*β* (siRNA^GSK-3*β*^)^39, 49, 60, 61^ in either cell type. The introduction of both a CT scrambled siRNA (siRNA^CT^) or siRNA^GSK-3*β*^ was first tested in pilot experiments by qPCR (Figure 6a) and western blotting (Supplementary Figure S5). In WT-N transfected with the siRNA^CT^, low dose of H_2_O_2_ induced an approximately 25% decrease in neuronal viability, which was associated with a small increase of caspase3-like activity, whereas GSK-3*β* knockdown followed by H_2_O_2_ treatment efficiently reversed caspase3 activation-induced cell death (Figures 6b and c). In TG-N transfected with a siRNA^C^^T^ and treated with PBS, AT8 protein levels were significantly higher when compared with their WT counterpart, whereas in GSK-3*β*-depleted TG-N, AT8 protein levels were significantly decreased (Figures 6d and e). These effects were associated with a decrease in DEVD-like fluorescent signal in siRNA^GSK-3*β*^-transfected TG-N, as opposed to TG-N treated with a scrambled siRNA (Figure 6c), thereby suggesting that TG-N death is associated with mHtt activation of pGSK-3*β*/pTau signaling promoting caspase3 activation.

### Depleting endogenous GSK-3*β* in TG astrocytes reverses Tau phosphorylation and caspase3 activation

Next the effect of GSK-3*β* depletion in TG astrocytes was addressed in the indirect co-culture paradigm. When TG-A were transfected with a siRNA^CT^ and layered on top of TG-N, a sharp loss of MAP2^+^ neurons was observed (Figure 6f), a decrease that was magnified when compared with the decreased MAP2+ neurons observed in TG-N alone. When cell death was assessed by caspase3 activation, a greater increase was detected in the TG-A/TG-N indirect co-cultures (Figure 6g) or TG astrocyte-conditioned media (ACM) paradigms (not shown) as compared with the TG-N alone, further supporting astrocyte-induced exacerbation of cell death. Interestingly, these effects were associated with a sharp upregulation of AT8 protein levels (Figures 6h and i) that was significantly greater as compared with TG-N without glia (Figures 6d and e). By contrast, GSK-3*β* depletion in TG-glia resulted in a significant reduction of caspase3 activation and AT8 upregulation in TG-N conditioned by TG-A transfected with a siRNA^CT^, together with a significant enhancement of MAP2^+^ neuronal loss (Figures 6f–m). These findings all suggest that the upregulation of GSK-3*β* in TG astrocytes may drive a Tau phosphorylation-induced increase in caspase3, thus exacerbating neuronal death in HD, at least in part, via mHtt-expressing astrocytes.

**Figure 7:**
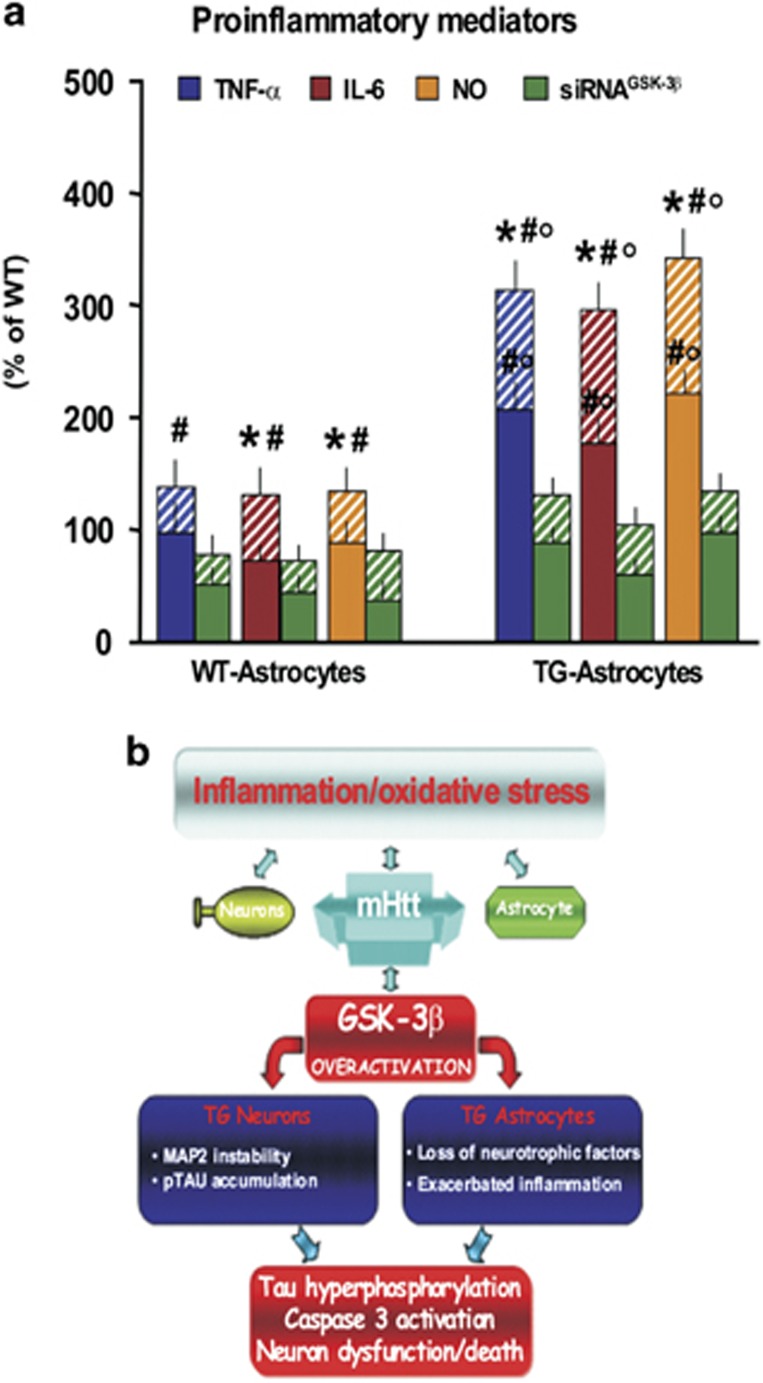
GSK-3*β* depletion counteracts cytokine overproduction of R6/2 astrocyte. (**a**) Astrocytes transfected with either a siRNA^C^^T^ or siRNA^GSK-3*β*^ were treated (dashed lines) or not with H_2_O_2_ (0.5 *μ*M) for 48 h. Levels of TNF-α and IL-6 in the medium were determined using an ELISA.^68, 70^ The level of NO in the medium was determined with the Griess reagent.^68, 70^ Data are presented as the mean±S.E. of three independent experiments. Statistical analysis was performed by one-way analysis of variance with Newman–Keuls Multiple Comparison *posthoc* test; *P<0.05 *versus* PBS within WT-A and TG-A cultures, ^#^*P*<0.01 *versus* WT; ^○^*P*<0.01 *versus* siRNA^GSK-3*β*^ within WT and TG astrocytes, respectively. (**b**) Astrocytes hyperexpressing GSK-3*β* drive Tau phosphorylation and caspase3 activation-induced cell death. Schematic drawing illustrating potential interactions of GSK-3*β* in mHtt-induced neuronal toxicity. mHtt-associated GSK-3*β* overactivation in hippocampal neurons may increase neuron vulnerability, promoting MAP2 instability and accumulation of pTau. Abnormal activation of GSK-3*β* in HD astrocytes may inhibit their neuroprotective functions and progressively exacerbates inflammation.^34, 35, 36, 37, 38, 39, 65^ All together and in synergy with mHtt-associated neuronal and astrocyte impairments, these effects may further magnify pTau accumulation leading to caspase3-dependent neuronal dysfunction/death

Finally, we assessed the levels of the proinflammatory cytokines, TNF-α and IL-6, and reactive nitrogen species production and found significantly higher levels in TG-A transfected with a siRNA^CT^ as compared with their WT counterparts (Figure 7a). Especially, H_2_O_2_ induced additional upregulation of the proinflammatory markers, and this was significantly attenuated by the depletion of GSK-3*β* (Figure 7a). Taken together, our findings show that active GSK-3*β* is upregulated in the HD hippocampus, both in neurons and astrocytes. This overactivation may potentiate the cytotoxicity of mHtt both directly and indirectly through its influence on astrocytes (Figure 7b).

## Discussion

We first analyzed GSK-3*β* transcription as a function of disease severity in HD postmortem hippocampal tissue and found that its transcriptional dysregulation is a prominent pathological feature of HD. Extensive immunohistochemistry analyses corroborated our findings by showing increased protein levels for GSK-3*β* and active pGSK-3*β*-Tyr^216^ in both HD hippocampal neurons and astrocytes as a function of disease severity. Coincident with this pathology, we also found phosphorylation of Tau at the AT8 epitope, as well as a decline in CV^+^/MAP2^+^ neurons in the DG of HD patients.

We then confirmed these findings in a well-recognized (R6/2) HD mouse model, recently shown to develop Tau hyperphosphorylation at multiple Tau phosphoepitopes (AT8, CP13, PT205 and PHF-1) following the onset of HD-like features.^21^ We found an upregulation of both pGSK-3*β*-Tyr^216^ and GFAP protein levels in the hippocampus of premanifest R6/2 mice that increased with disease progression. This suggests that mHtt may activate GSK-3*β* both at an early stage of the disease process as well as throughout the disease course. Thus we chose to explore this further *in vitro* with experiments, which revealed that there is an upregulation of pGSK-3*β*-Tyr^216^ in both primary neurons and astrocytes harvested from the R6/2 mouse brain as well as decreased R6/2 neuronal viability. This, we hypothesized, could be due to an elevated level of oxidative stress. To confirm this, we mimicked such a state by challenging TG-N with a moderate oxidative stimulus and by using a specific GSK-3*β* antagonist as well as genetic depletion of GSK-3*β*, and we were able to causally link active GSK-3*β* to mHtt-induced increased AT8 accumulation, caspase3 activation and R6/2 neuronal cell loss. To further dissect the potential role of R6/2 astrocytes in this process, we used different co-culture paradigms between WT/TG astrocytes and WT/TG neurons. This revealed that TG-A overexpressing GSK-3*β* lose their neurotrophic and neuroprotective properties and thus fail to protect and support either WT or TG hippocampal neurons, via, in part, the aberrant production of proinflammatory mediators. Furthermore, depletion of GSK-3*β* in R6/2 astrocytes showed that mHtt-induced GSK-3*β* overexpression in this cell type is also a crucial factor driving AT8 accumulation, caspase3 activation and reduced neuronal survival. All of this suggests that GSK-3*β* overactivation may potentiate mHtt neurotoxic effects both directly and indirectly through an influence on astrocytes (Figure 7b).

Converging evidence implicates GSK-3*β* as a key signaling molecule involved in the maintenance and function of adult neurons.^25, 26, 27, 28, 29, 30, 31, 32, 33, 38, 39, 40, 43, 44, 45, 48, 49, 50, 59^ In particular, GSK-3*β* activation has a central role in regulating the neuroinflammatory and astroglial response to neurodegeneration.^35, 36, 37, 38, 39^ There is also mounting evidence to show that mHtt impairs astrocyte properties and that this may have an important role in the pathology of HD.^5, 13, 14, 15, 16, 17, 18^ Taken together, these findings suggest that GSK-3*β* dysregulation in both neurons and glia represents a crucial vulnerability factor and a potential target for mitigating the progression of pathology in HD hippocampus.

A special feature of GSK-*β* in neurodegenerative diseases is the phosphorylation of the protein Tau, which is detrimental to neurons. Recently, pathological Tau aggregates have been seen in cortical and striatal HD tissue,^22, 23^ and while our new study did not specifically address this aspect of HD pathogenesis, we did observe AT8 neuronal inclusions in the GCL and hilus of Grades 2–3 hippocampal sections from HD patients. In animal models of HD, Gratuze *et al.*,^21^ have also recently reported that there is extensive phosphorylation of Tau in the hippocampus, cortex and striatum of R6/2 and 140 CAG knock-in mice, subsequent to the onset of HD pathology,^21^ and the present study corroborates this by showing increased AT8 protein levels in the R6/2 hippocampus. The critical role of dysregulated endogenous GSK-3*β* in this process was further documented in GSK-3*β*-depletion experiments, where GSK-3*β* knockdown in TG-N significantly attenuated caspase3 activation and AT8 accumulation. Moreover, we show that there is an additional contribution to this cell loss from changes in GSK-3*β* in mHtt-containing astrocytes, supporting astrocyte–neuron interactions as essential components in the neuronal dysfunction that characterises neurodegenerative diseases, including HD.^5, 13, 14, 15, 16, 17, 18, 38, 39, 62, 63, 64, 65^

This enhanced GSK-3*β* activation in TG-A not only impaired their neurotrophic and neuroprotective properties but also significantly contributed to mHtt-induced Tau phosphorylation and caspase3 activation, possibly via an exacerbated production of major proinflammatory mediators (Figure 7), as recently highlighted in the study of Garwood *et al.*^64^ indicating that astrocytes are important for A*β*-induced Tau phosphorylation, caspase3 activation and cell death.^64^

In conclusion, we have shown that there is mHtt-induced GSK-3*β* dysregulation in both neurons and astrocytes of the hippocampus in HD has a crucial role in the evolving pathology seen in this disorder. This may not only explain the selective vulnerability of this structure to the disease process and its clinical expression but also open up new therapeutic avenues by which to slow down or even reverse this currently incurable condition.

## Materials and Methods

### Study design

The aim of the postmortem work was twofold: (i) to investigate the expression and impact of GSK-3*β* and its active pGSK-3*β*-Tyr^216^ isoform in the HD hippocampus at different stages of disease; and (ii) to identify reliable endogenous candidate reference genes for normalization of qPCR targeted gene expression in human HD hippocampal tissue. The study was approved by the Ethical Research Committee of the Centre Hospitalier Universitaire de Québec (#A13-02-1138) and the OASI Institute for Research and Care on Mental Retardation and Brain Aging (Troina, EN, Italy) ethical board following the guidelines of the Declaration of Helsinki. Human brain tissues were obtained from the Cambridge Brain Bank and used under full ethical approval (REC 01/177).

### Postmortem samples

HD cases (CAG>35) ranged from 43 to 78 years of age (56.8±12) years (Table 1). All diagnoses were based on genetic confirmation of the expanded CAG repeat (see Table 1) along with clinical history and histopathological evaluation by an experienced neuropathologist using the Vonsattel classification.^53^ Sample distribution included Grade 2 (*n*=5), Grade 3 (*n*=7) and Grade 4 (*n*= 10) tissues; the mean sizes of the longer and shorter CAG repeats were 48.0±5.5 and 19.0±1.4, respectively. The postmortem interval (PMI) was 25.7±12.4 h and brain acidity–alkalinity (pH) values were 6.54±0.3. CT samples (*n*=11) were neurologically normal cases, matched for age (65.1±14 years), PMI (26.4±9.9 h) and pH values (6.49±0.38). Fresh frozen dissected hippocampi were used for RNA isolation while paraffin-embedded sections were used for immunofluorescence analyses.

### Human reference gene panel

A variety of factors affect reference gene mRNA in postmortem human and rodent brain tissues and a careful and stringent selection of a proper constitutively expressed CT gene prior to qPCR analysis is therefore essential.^54, 55, 56, 66^ We thus analyzed 19 candidate genes (Human Reference Gene Panel, Roche Applied Bioscience, Penzberg, Germany, http://www.roche-applied-science.com) from different abundance and functional classes, derived from literature searches and as presented in Supplementary Table S1.

### RNA isolation, cDNA synthesis and qPCR assays

The human hippocampus was dissected (approximately 50 mg) using a cryostat and collected for RNA extraction using an RNeasy Lipid Tissue Mini Kit (Qiagen, Toronto, Ontario, Canada) and RNA extraction procedure carried out as described.^67^ Briefly, hippocampal tissue was homogenized in 1 ml of trizol, followed by the addition of 200 *μ*l of chloroform. The homogenate was subsequently separated into aqueous and organic phases by centrifugation and ethanol was added to the aqueous phase before being passed through an RNeasy column. RNA was eluted in 30 *μ*l of RNAse-free water. The purity of the RNA was determined by measuring the absorbance A260/A280 in a buffered solution at pH 7.4 using nanodrop. The quality and integrity of the extracted RNA was assayed by agarose gel electrophoresis to detect the presence of ribosomal RNA degradation.^67^ One *μ*g of RNA was taken for synthesis of complementary DNA (cDNA) using a cDNA Synthesis Kit (Life Technologies Europe BV, Monza, Italy, www.lifetechnologies.com) according to the manufacturer's guidelines. The qPCR was carried out as described^38, 39, 60, 61^ (Supplementary Experimental Procedures), using 50 ng of cDNA and according to the manufacturer's protocol (http://www.roche-applied-science.com).

### Gene analysis and statistics

To compare gene expression stability and rank, geNorm,^54^ NormFinder^55^ and BestKeeper^56^ algorithms were used as detailed in Supplementary Methods. To maximize power, analyses were applied to three groups: (i) all HD cases combined with the neurologically normal cases (CT) as a single group (A); (ii) the HD group alone (B) and (iii) the CT group alone (C). Non-paired Student's *t*-tests were used to determine whether the CT and HD groups differed on individual reference genes or geomean. To compare the homogeneity of the qPCR values of the 33 samples, we performed a two-sided Grubbs' test (at significance level *P*≤0.01) for each gene. Correlations among continuous variables were performed to determine whether any of the studied variables related to housekeeper/target gene mRNA levels or their geometric means. Statistical significance level was set at *P*≤0.05.

### Target gene studies

After combinatorial analyses for the selection of a suitable reference gene and validation by comparison with less suitable ones (PPIA, S18) using quantification of the GFAP gene, we addressed changes in mRNA levels of GSK-3*β* using RPLP0 as the internal CT gene (Supplementary Figure S3). We used a duplicate assay for each sample. GSK-3*β* (ID: Hs01047719-m1), GFAP (ID: Hs00909233-ml), RPLP0 (ID:4326314E), PPIA (ID: 4326316E) and S18 (ID: 4319413E) were obtained from Applied Biosystems (Life Technologies). Quantification of the abundance of target gene expression was determined relative to the reference gene with respect to the CT group by using the delta delta Ct (2^−^^ΔΔCt^) comparative method. Statistical analysis of variance used a one-way ANOVA, followed by correction for multiple testing using the Tukey's Multiple Comparison test.

### Immunofluorescence and microscopy analyses

Hippocampal slide series of HD (*n*=16) as well as age- and sex-matched CT (*n*=10) cases were studied. Immunofluorescent staining was carried out on serial paraffin-embedded hippocampal tissue sections (6 *μ*m in thickness). Adjacent sections were stained with CV acetate (0.1 w/v) to count the number of neurons present in the GCL in each section.^38, 39^ Immunostaining was carried out as described previously.^60, 61, 67, 68, 69^ Briefly, after deparaffinization, antigen retrieval was performed by incubating the sections in citrate buffer 10 nM pH 6 at 95 °C for 30 min. The sections were subsequently washed three times in PBS at room temperature (RT). To reduce signal interference from tissue autofluorescence, all sections were incubated with Sudan Black B (Harleco, Philadelphia, PA, USA), 0.5% in 70% alcohol, for 5 min. Sections were washed in PBS containing 0.2% Triton X-100 (3 × 10 min) between each step. Primary and secondary antibodies (Abs; Supplementary Table S4) were diluted in a blocking buffer of 5% normal serum, 5% bovine serum albumin and 0.4% Triton X-100 in PBS. After overnight incubation with the primary Abs, the sections were rinsed and incubated in darkness for 2 h with CY3/FITC-conjugated donkey anti-rat, donkey anti-rabbit, donkey anti-mouse and donkey anti-goat antibodies (1 : 200–400; Jackson ImmunoResearch, Suffulk, Europe LTD, UK), mounted on glass slides and coverslipped with glycerol-based mounting medium. All Abs used in this study have been previously tested and validated for immunofluorescence and western blotting.^38, 39, 60, 61, 62, 70^ Nuclei were counterstained with 4′,6-diamino-2-phenylindole (DAPI) in mounting medium, (H1200) Vector Laboratory, Burlingame, CA, USA.

Slides where the primary antibody was omitted served as a negative CT to ensure that there was no autofluorescence from the secondary antibody in the reaction sequence of the labeling experiments. Immunostaining was examined using an Olympus fluorescent microscope BX-51 (Segrate, Milan, Italy) and a Leica TCS-SPE confocal microscope (Leica Lasertechnik GmBH, Heidelberg, Germany).

### Confocal laser scanning microscopy, image analysis and quantification of immunostaining

All assessments were performed by a blinded observer. For fluorescent microscopy of hippocampal sections, images were obtained using an inverted Leica LCS-SPE confocal microscope (Leica Lasertechnik GmBH) equipped with an argon/krypton laser or a Fluoroview FV1000 confocal microscope (Olympus Canada Inc., Richmond Hill, Ontario, Canada). For FI assessments and co-localizations, hippocampal sections were labeled by immunofluorescence, and images were acquired by sequential scanning of 12–16 serial optical sections. 3D reconstructions from *z*-series were used to verify co-localization in the *x*–*y*, *y*–*z* and *x*–*z* planes. To estimate cellular pGSK-3*β*/-Tyr^216^ fluorescence in the DG, the cell fluorescence was acquired separately with the FITC and CY3 filters, as detailed elsewhere.^60, 69^ For cell counting, serial fluorescent images were captured in randomly selected areas along the DG and the number of labeled cells per field (14–18 fields/section) was manually counted in three to four hippocampal sections/case, using the Olympus cellSense Dimension software (Segrate, Milan, Italy), and results expressed as mean±S.D.

### Studies in R6/2 mice

R6/2 mice, TG for exon 1 of the human Htt gene^58^ and WT littermates were initially obtained from the Jackson Laboratory (Bar Harbor, ME, USA) and mated with female CT mice (B6CBAFI/J). Offspring were verified by a PCR genotyping technique using DNA extracted from tail tissues using primers (5′-CCGCTCAGGTTCTGCTTTTA-3′ and 5′-GGCTGAGGAAGCTGAGGAG-3′) located in the transgene. The study was carried out in premanifest (3 weeks of age) and manifest mice (>8 weeks of age) as the onset of HD features in these mice occurs at 8 weeks of age.^58^ The tail test was used to detect the abnormal clasping of the hind limbs and was employed to ensure the premanifest status of the R6/2 mice.^58^ Mice were suspended by the tail for 10 s; if the mouse acquired a locked body position, the result was scored as positive. The animals were weighed once a week and handled according to National Institutes of Health guidelines. The protocol received approval by the institutional Animal Care and Use Committee, and all studies were conducted in accordance with the United States Public Health Service's Policy on Humane Care and Use of Laboratory Animals.

### Primary neuronal cell cultures

Primary cultures of hippocampal neurons were generated from TG R6/2 and WT mice. Briefly, at embryonic day 17, tails from each embryo were used to extract genomic DNA and to perform PCR for genotype determination. The brains were next removed and hippocampi were dissected free of meninges in Ca2^+^/Mg^2+^-free Hanks' balanced salt solution (HBSS) and rinsed twice with HBSS allowing the tissue to settle to the bottom of the tube. After the second wash, the tissue was resuspended in HBSS containing 0.25% (w/v) trypsin (without phenol red) and incubated for 15 min at 37 °C. After three rinses with HBSS, the tissue was mechanically dissociated in plating medium (Dulbecco's modified Eagle's medium, GIBCO, Rockville, MD, USA), supplemented with 10% horse serum (GIBCO), 100 U/ml penicillin and 100 mg/ml streptomycin by gentle passage through Pasteur pipettes. Dissociated hippocampal cells were seeded in poly-L-lysine-coated six-well culture plates at a density of 7 × 10^5^ cells per well in plating medium. Cultures were maintained at 37 °C in 5% CO_2_ for 2 h before the plating medium was replaced with neurobasal growth medium (GIBCO) supplemented with B27 (GIBCO), 2 mM L-glutamine, 100 U/ml penicillin and 100 mg/ml streptomycin. At day 2, cultured neurons were treated with 1-*β*-D-arabinofuranosylcytosine 2 *μ*M for 24 h.^38, 39^ The medium was changed every 3 days, and after 7–10 days in culture (days *in vitro* (DIV)), cells were used for the experiments. The purity of the neuronal cultures (≥95%) was determined by immunofluorescence staining for the neuron-specific marker, MAP2.

### Primary astrocyte cultures

Primary astrocyte cell cultures were obtained from mouse hippocampi of postnatal 2–3-day-old WT and TG mice, as described previously.^38, 39, 60, 61, 69, 71^ The cultures were allowed to grow and differentiate until they reached confluency at which time (21–25 DIV) the loosely adherent microglial cells were separated by shaking for 2 h at 37 °C and 190 r.p.m. The attached cells were then washed with sterile PBS and incubated for 1–2 h at 37 °C incubator, at 5% CO_2_, before overnight shaking at 37 °C and 210 r.p.m. The supernatant media containing oligodendrocyte precursors and other cell types were discarded. The glial (>95% of the cells were GFAP-IR astrocytes) monolayers were then rinsed with sterile PBS and replated at a final density of 0.4–0.6 × 10^5^ cells/cm^2^ in poly-D-lysine (10 *μ*g/ml)-coated 6, 12- or 24-well plates or in insert membranes (0.4 *μ*m polyethylene terephthalate) for indirect co-culture (BD Biosciences, Becton Dickinson Labware, Franklin Lakes, NJ, USA). Astrocyte monolayers were used for direct or indirect co-cultures or processed for gene silencing and used for indirect co-cultures with primary hippocampal neurons, as described. Alternatively, the neuronal cultures were exposed to the ACM.^69^

Preliminary dose–response and time course pilot experiments on neuronal and astrocyte cell viability of WT and TG cultures were first carried out using a range of concentrations of H_2_O_2_ (0.25–25 *μ*M) and a concentration range of 0.5 *μ*M was selected for the study. Consistent with earlier and more recent reports on primary cortical and striatal R6/2 cultures, we detected an increased vulnerability of hippocampal R6/2 neurons, while the viability of R6/2 astrocytes was not affected by such stressors^13, 17, 18, 40^ (Supplementary Figure S4). Both purified neuronal cultures and astroglial–neuronal co-cultures at 8–9 DIV received PBS or H_2_O_2_ (0.5 *μ*M). Pharmacological antagonism of GSK-3*β* was carried out with the specific GSK-3*β* antagonist, AR (of 5 *μ*M)^38, 39^^,71^ administrated for 60 min before H_2_O_2_. Viability assays, caspase3 activity and protein extracts for western blotting determinations of protein levels were carried out as part of these neuronal cultures; other neuronal cultures were used for immunocytochemistry and cell counting. Briefly, the cultures were fixed in 4% paraformaldehyde in PBS and processed using immunofluorescent staining procedures,^38, 39, 52, 53^ employing anti-MAP2 and anti-GFAP, as described above. Nuclei were counterstained with DAPI. Analyses were performed using a confocal laser microscope and computer-assisted image analysis (Leica). For quantification of the amount of cells expressing a given marker or marker combinations, the number of MAP2^+^ cells was determined relative to the total number of DAPI-labeled nuclei, using the Leica lite Software (Leica, Heidelberg, Germany) and three-dimensional overlay to avoid false-positive/negative overlay and double counting.

### Cell viability and caspase3 activity assays

Primary neuronal and astrocyte cell cultures treated as above were analyzed for membrane integrity by exclusion of Trypan blue (0.12% wt/vol), metabolic reductase activity using the MTT assay and cell death by the LDH release and caspase3 assay.^38, 39, 60, 61, 69^ For the MTT assay, MTT was added to the existing culture medium (final concentration of 120 *μ*M) for 15 min after which the media was removed. Reduced MTT solubilized with 200 *μ*l DMSO was then measured by absorbance at 585 nm. The LDH assay used the LDH Cytotoxicity Detection Kit (Roche) as per the manufacturer's instructions. For caspase3 activity measurements, the cells were lysed in ice-cold lysis buffer and processed as detailed elsewhere.^38, 39, 60, 61, 69^ The fluorogenic substrate DEVD-AFC (15 *μ*M; Calbiochem System Products, San Diego, CA, USA), used for quantification of DEVD-like fluorescent signal, was assessed using a luminescence spectrophotometer (Beckman Coulter, Cassina de' Pecchi, Milan, Italy) (excitation 400 nm and emission 505 nm). Enzymatic activity (arbitrary fluorescent units) is expressed as the percentage of CTs.

### Transient gene silencing with siRNA

To test the effect of GSK-3*β* protein depletion, we used targeted mRNA degradation using siRNA performed as described previously.^39, 49, 60, 61^ GSK-3*β* siRNA (sc-35525) and CT siRNA (sc-37007) were purchased from Santa Cruz Biotechnology (Dallas, TX, USA). siRNA introduction was performed according to the protocol provided by Santa Cruz Biotechnology, as reported in detail in our previous studies.^39, 60, 61^ Briefly, to prepare lipid–siRNA complexes, 80 pmol of the indicated siRNA duplex in 100 *μ*l of Transfection medium (sc-36868) and 6 *μ*l of siRNA Transfection reagent (sc-29528) in 100 *μ*l of Transfection medium were combined, incubated for 30 min at 25 °C and diluted with 800 *μ*l of prewarmed Transfection medium. The cells were dissociated and plated on 24 plate cells in growth medium, rinsed once with serum-free DMEM and 1000 *μ*l of lipid–siRNA mixture (as described above) was applied to each well. After incubation for 6 h at 37 °C in a humidified 5% CO_2_ cell culture chamber, an additional 1 ml of 20% FBS in DMEM was added to each well, and lipofection was allowed to continue overnight. The next morning, the lipofection medium was aspirated, and transfected cells were re-fed with fresh growth medium. The cells were collected 72 h after transfection for qPCR and western blotting assays.

### Protein extraction and western blotting

For hippocampal tissues, mice were killed by decapitation and the brains immediately removed and the tissues dissected on ice, then frozen on dry ice and kept at −80 °C until studied. Dissected hippocampi were homogenized and processed, as described previously^38, 39^ (Supplementary Methods), proteins quantified using the BCA protein determination method (Bio-Rad, Hercules, CA, USA). SDS-PAGE and western blotting analysis of hippocampal samples/cell homogenates were then performed as detailed in L'Episcopo *et al.*^61^ and Tunbridge *et al.*^66^ Briefly, hippocampal tissue or cell homogenates were separated on SDS-10% polyacrylamide gel and then transferred onto nitrocellulose membranes (Amersham Biosciences, Pittsburgh, PA, USA). Non-specific binding sites were blocked with 5% non-fat dry milk in phosphate-buffered saline containing 0.1% Tween 20 (PBS-T) for 1 h at RT and were afterwards incubated overnight at 4 °C with the specific antibodies (Supplementary Table S4). The following day, membranes were washed three times in PBS-T and then incubated for 1 h at RT with the corresponding HRP-conjugated secondary antibodies in 5% non-fat dry milk in PBS-T, and the immunoreactive signal intensity was visualized by enhanced chemiluminescence (ECL Plus, GE Healthcare Biosciences, Piscataway, NJ, USA). Immunoreactive bands were visualized using the ImageQuant LAS 4000 imaging system (Fujifilm USA, Valhalla, NY, USA). Quantification of phosphoepitopes was performed relative to their non-phosphorylated proteins and was normalized to *β*-tubulin or *β*-actin as loading controls. Results are expressed as the percentage of WT mice. Data are mean±S.D. with *n*=6 for each condition.

### Enzyme-linked immunosorbent assay (ELISA) and NO production

Levels of cytokines were determined using the ELISA kits (DuoSet ELISA Development System, R&D Systems, McKinley Place, MN, USA) following the manufacturer's protocol.^60, 72^ NO production was determined by measuring the accumulated levels of nitrite in the culture supernatants with the Griess reagent.^61, 72^

### Statistical analyses

Results for immunocytochemistry, immunoblots, *in vitro* viability and caspase3 assays, GSK-3*β* depletion experiments and ELISA assays are expressed as mean±S.D. unless otherwise noted. Statistical analysis was performed by one-way ANOVA with Newman–Keuls Multiple Comparison *posthoc* test. Statistical significance was accepted at *P*<0.05.

## Figures and Tables

**Table 1:** Characteristics of the human hippocampal samples

**ID**	**Grade**	**Sex**	**Age**	**CAG allele1**	**CAG allele2**
HD1	2	M	73	21	45
HD2	2	M	64	20	45
HD3	2	M	66	19	46
HD4	2	F	78	17	43
HD5	2	M	45	18	47
HD11	3	M	54	23	47
HD12	3	F	61	17	46
HD13	3	M	55	19	51
HD15	3	M	59	19	46
HD16	3	F	65	17	44
HD17	3	M	78	19	43
HD18	3	M	73	21	45
HD21	4	M	44	18	53
HD22	4	M	43	21	53
HD23	4	M	40	18	51
HD24	4	M	47	20	52
HD25	4	M	50	19	50
HD26	4	M	65	18	46
HD27	4	F	26	22	70
HD28	4	M	43	18	51
HD29	4	M	51	20	52
HD30	4	F	53	20	52
HD31	4	M	68	19	45

**ID**	**Sex**	**Age**			
CT1	M	75			
CT2	M	69			
CT3	M	61			
CT4	M	67			
CT5	M	67			
CT6	F	63			
CT7	F	61			
CT8	M	59			
CT9	F	72			
CT10	M	67			
CT11	M	56			
AD1	M	76			

## Abbreviations

ACM: astrocyte-conditioned media
AR: AR-AO14418
AT8: phospho-PHF-Tau
CV: cresyl violet
DAPI: 4′,6-diamino-2-phenylindole
DG: dentate gyrus
DIV: days *in vitro*
FI: fluorescence intensity
GCL: granule cell layer
GFAP: glial fibrillary acidic protein
GSK-3*β*: glycogen synthase kinase-3*β*
pGSK-3*β*-Tyr^216^: phosphorylated GSK-3 beta at Tyrosine 216
HBSS: Hanks' balanced salt solution
HD: Huntington's disease
H_2_O_2_: hydrogen peroxide
Htt: huntingtin protein
LDH: lactate dehydrogenase
MAP2: microtubule-associated protein 2
MAPT: microtubule-associate protein Tau
mHtt: mutant huntingtin protein
MTT: 3-(4,5-dimethylthiazolyl-2)-2,5-diphenyltetrazolium bromide
PBS-T: phosphate-buffered saline containing Tween 20
PMI: postmortem interval
polyQ: polyglutamine
qPCR: quantitative PCR
siRNA: small interfering RNA
TG: transgenic
WT: wild type
