# Supplementary material for: GSK-3β-induced Tau pathology drives hippocampal neuronal cell death in Huntington's disease: involvement of astrocyte–neuron interactions
Source: Cell Death Dis. 2016 Apr 28;7(4):e2206–. doi: 10.1038/cddis.2016.104 (PMC4855649; doi:10.1038/cddis.2016.104)
Supplement: Supplementary Information [file cddis2016104x1.doc]

Revision Manuscript CDDIS-16-0033-T

**Supplemental Informations**

**GSK-3β-induced Tau pathology drives hippocampal neuronal cell death in Huntington’s disease: involvement of astrocyte-neuron interactions**

Francesca L’Episcopo, Janelle Drouin-Ouellet, Cataldo Tirolo, Alfredo Pulvirenti, Rosalba Giugno, Nunzio Testa, Salvatore Caniglia, Maria Francesca Serapide, Giulia Cisbani, Roger A. Barker, Francesca Cicchetti & Bianca Marchetti

**Inventory of Supplemental Information**

Figure S1

Figure S2

Figure S3

Figure S4

Figure S5

Supplemental Figure Legends

Table S1, Selected candidate reference genes for gene expression analysis

Table S2, Gene stability value calculation by normFinder

Table S3, Gene coefficient variation calculation by BestKeeper

Table S4, Antibody used in immunoblot and immunofluorescent staining analyses

Supplemental Experimental Procedures

Author Contributions

Acknowledgements

**Supplemental Figure Legends**

**Supplementary Figure 1**. **Expression levels of selected endogenous reference genes in neurologically normal CT and HD hippocampal samples.** qPCR was carried out on a total of 11 CT and 22 HD hippocampal samples. Nineteen endogenous reference genes were selected (Table S1). Case IDs are indicated, and their details are reported in Table 1. The cycle threshold (Ct) value (mean ± SD) is plotted against each gene cluster. A broad panel of Ct values is observed. Both in the CT (A-B) and HD (C-E) samples, *18S rRNA* gene showed the lowest mean Ct value indicating abundant mRNA levels, while *GUSB* had the highest mean Ct values and lowest abundance. Nonpaired Student’s *t*-tests indicated that none of the variables studied affected reference gene mRNA levels nor their geometric mean within the control and HD samples, separately. By contrast, in HD, a significant (**p* < 0.05) decrease of Ct was observed as compared to Control cases for the following genes: *ACTB*, *GAPDH*, *ALAS*, *B2M*, *GUSB*, *PGK1* and *PPIA.* No outliers were detected in the CT group by the Grubbs’ test, whereas in Grade 4 HD (E), HD23 was identified as an outlier (**p* < 0.01), and thus excluded from further analyses.

**Supplemental Figure 2. GeNorm analysis of the (A) total sampled population, (B) HD cases and (C) Control cases alone.** Average expression stability (M) of 8 candidate reference genes and the best combination of 2 genes (A-C). Determination of the optimal number of reference genes for normalization was undertaken (D, E, and F). The geNorm program calculated the normalization factor (NF) from at least 2 genes and the variable V defines the pair-wise variation between the 2 sequential NFs. *RPLP0*/*RPL13A* are the most stable reference genes with the lowest M values, whereas *S18* and *B-globin*, were the least stable genes.

**Supplemental Figure 3. Validation of *RPLP0* against *18S* and *PPIA* for normalization of GFAP relative gene expression and immunofluorescence analyses in HD and CT hippocampal samples.** **A**: relative mRNA expression of the target molecule GFAP in a total of 20 hippocampal tissues from HD and CT matched cases (n = 5 samples/case). Statistical differences in mRNA expression levels (Mean ± SD) were determined using a one-way ANOVA followed by correction for multiple testing using the Tukey’ Multiple Comparison test. *GFAP* mRNA varied markedly when normalized to the least stable reference genes (*18S* or *PPIA).* By contrast, the normalization with *RPLP0* reduced the inter-individual variability within HD and CT sampled groups; ***p* < 0.001 vs CT. **B**: GFAP fluorescence intensity (FI) and GFAP^+^ cell number/field in DG areas (Means ± SD) as assessed in hippocampal sections from CT (n= 5) and HD (n= 5) cases corroborates increased GFAP expression in HD (***p* < 0,001 vs. CT). **C-D**: Representative images of hippocampal sections showing GFAP-IR (C, green) in the DG of a control, a Grade 3, and a Grade 4 (D, red). Note the arrangement of GFAP^+^ astrocytes at the base of the granule cell layer (GCL) within the subgranular zone (SGZ) of the DG, with their typical morphology of small cell bodies and thin GFAP^+^ processes extending across the granule cell layer (GCL). By contrast, this organization was not evident in HD (D) and the astrocytes displayed a reactive phenotype with increased somal size with thickened and shorter processes (D).

**Supplemental Figure 4**. **The vulnerability** **of R6/2 hippocampal neurons but not R6/2 astrocytes is increased in basal conditions and after oxidative stress.** Cytotoxicity assays were carried out in enriched primary hippocampal neurons (neurons alone) at 7-10 days *in vitro* (DIV) and primary astrocyte (astrocytes alone) cell cultures at 25 DIV, both after PBS or a mild oxidative stress challenge (H_2_O_2_, 0,5 µM) in WT and TG cultures. Cell viability was assessed by exclusion of Trypan blue (0.12% wt/vol) (A) and caspase3-like activity (B) using the fluorogenic substrate DEVD-AFC (see Method section). Values are expressed relative to untreated WT cells (% of WT) and represent the mean ± SD of 4 replicates. * p < 0,05 vs. PBS in WT and TG cultures respectively; ** p < 0,01 vs WT cultures. **Abbreviations**: H_2_O_2_: hydrogen peroxide; TG, transgenic; WT, wild type.

**Supplemental Figure 5.**  **GSK-3β silencing-induced depletion of GSK-3β protein levels in TG neurons and TG astrocytes.** Cells were transfected with scrambled siRNA (siRNA^Ct^) or GSK-3β specific siRNA (siRNA ^GSK-3β^) as described in Materials and Methods, and 72 h posttransfection, cells lysates were analyzed for total GSK-3β immunoreactivity by Western blotting (A-B). Blots were reprobed with the anti-β-tubulin antibody. The expression level of GSK-3β was estimated by densitometry analyses as described in the Method section and results (A) are expressed as percentage of control (siRNA^Ct^). Data are mean ± SD with n= 5 for each condition. Two representative continuous lanes are displayed (TG-N; TG-A) after transfection with a scrambled siRNA^Ct^ or siRNA ^GSK-3β^. Statistical differences were analyzed by ANOVA followed by a Newman-Keuls test. * *p* < 0,01 vs. siRNA^CT^ within TG-N and TG-A, respectively. GSK-3β, Glycogen synthase kinase 3 beta; N, neurons; A, astrocytes; TG, transgenic; WT, wild type.

**Table S1. Selected candidate reference genes for gene expression analysis**

| **Abbreviation** | **Gene** | **Cellular function** |
| --- | --- | --- |
| 18S rRNA | 18S ribosomal RNA | ribosome subunit |
| ACTB | β-actin | cytoskeleton |
| ALAS | 5-aminolevulinate synthase | mitochondrial precursor |
| B2M | β-2-microglobulin | major histocompatibility complex |
| β-globin | hemoglobin, beta | structure of polypeptide chains in adult hemoglobin |
| G6PDH | glucose-6-phosphate dehydrogenase | glycolysis enzyme |
| GAPDH | glyceraldehyde-3-phosphate dehydrogenase | glycolysis enzyme |
| GUSB | β-glucuronidase | glycosaminoglycan degradation |
| HPRT1 | hypoxanthine ribosyltransferase | metabolic salvage of purines |
| IPO8 | importin 8 | intracellular protein transport |
| PBGD | hydroxymethylbilane synthase | enzyme of the heme biosynthetic pathway |
| PGK1 | phosphoglycerate kinase 1 | glycolytic enzyme |
| PPIA | peptidyl-prolyn cis-trans isomerase A | folding of proteins |
| RPLP13A | ribosomial protein L13a | ribosome |
| RPLPO | ribosomal phosphoprotein, large, P0 | ribosome |
| SDHA | succinate dehydrogenase complex, subunit A, flavoprotein (Fp) | mitochondrial respiratory chain |
| TBP | TATA-box-binding protein | transcription factor |
| TFRC | transferrin receptor | cellular iron uptake |
| YWHAZ | tyrosine 3-/tryptophan 5-monooxygenase-activation protein, zeta isoform | signal transduction |

**Table S2. Gene stability value calculation by normFinder**

| **Gene** | **Stability value 1** | **Gene** | **Stability value 2** | **Gene** | **Stability value 3** |
| --- | --- | --- | --- | --- | --- |
| **RPL13A** | 0,005 | **RPL13A** | 0,007 | **RPLP0** | 0,003 |
| **RPLP0** | 0,008 | **RPLP0** | 0,012 | **RPL13A** | 0,007 |
| **PGK1** | 0,018 | **TFRC** | 0,016 | **TFRC** | 0,020 |
| **PBGD** | 0,019 | **G6PDH** | 0,021 | **PGK1** | 0,021 |
| **TFRC** | 0,024 | **PGK1** | 0,028 | **G6PDH** | 0,031 |
| **G6PDH** | 0,024 | **PBGD** | 0,034 | **β-globin** | 0,037 |
| **β-globin** | 0,038 | **β-globin** | 0,040 | **PBGD** | 0,056 |

**Table S3. Gene coefficient variation calculation by BestKeeper**

| **Pooled**  **groups** | **R** | **CV** | **SD** | **HD**  **cases** | **R** | **CV** | **SD** | **Controls** | **R** | **CV** | **SD** |
| --- | --- | --- | --- | --- | --- | --- | --- | --- | --- | --- | --- |
| **RPL13A** | 0,951 | 3,14 | 0,84 | **RPL13A** | 0,96 | 3,01 | 0,8 | **RPLP0** | 0,892 | 2,57 | 0,72 |
| **RPLP0** | 0,927 | 3,03 | 0,84 | **RPLP0** | 0,938 | 2,93 | 0,81 | **β-globin** | 0,532 | 2,41 | 0,72 |
| **TFRC** | 0,957 | 4,15 | 1,25 | **PBGD** | 0,913 | 2,83 | 0,91 | **RPL13A** | 0,942 | 3,41 | 0,93 |
| **PBGD** | 0,426 | 4,04 | 1,27 | **G6PDH** | 0,877 | 3,21 | 1 | **TFRC** | 0,97 | 3,93 | 1,21 |
| **β-globin** | 0,775 | 4,17 | 1,28 | **PGK1** | 0,952 | 3,85 | 1,09 | **G6PDH** | 0,916 | 4,08 | 1,3 |
| **PGK1** | 0,923 | 4,76 | 1,36 | **β-globin** | 0,727 | 3,74 | 1,13 | **PBGD** | 0,762 | 4,31 | 1,41 |
| **G6PDH** | 0,821 | 4,34 | 1,37 | **TFRC** | 0,952 | 3,95 | 1,19 | **PGK1** | 0,856 | 5,24 | 1,53 |

**Table S4. Antibody used in immunoblot and immunofluorescent staining analyses.**

| **Primary Ab** | **Source** | **Product** | **Application** | **Dilutions** |
| --- | --- | --- | --- | --- |
| GSK-3β | BD Transduction | 610202 | IB, IF | 1:1000 (IB), 1:200 (IF) |
| p-GSK-3β-Tyr^216^ | BD Transduction | 612313 | IB, IF | 1:1000 (IB), 1:200 (IF) |
| GSK-3β | Santa Cruz | Sc:9166 | IB, IF | 1:1000 (IB),1:200 (IF) |
| p-GSK-3β-Tyr^216^ | Santa Cruz | Sc-135653 | IB, IF | 1:1000 (IB), 1:200 (IF) |
| MAP2 | Millipore | AB5622 | IF | 1: 300 (IF) |
| MAP2 | Chemicon | MAB3418 | IF | 1: 300 (IF) |
| GFAP | Sigma Aldrich | C9205 | IF | 1: 400 (IF) |
| GFAP | Dako Cytomation | Z0334 | IF | 1:400 (IF) |
| GFAP | Millipore | AB5541 | IB | 1:1000 (IB) |
| PHF-tau pSer202/Thr205, Clone AT8 | Thermo Scientific | MN 1020 | IB, IF | 1:1000 (IB), 1:200 (IF) |
| Tau-5 | Millipore | MAB361 | IB | 1:1000 |
| β-tubulin Tub 2.1 | Sigma Aldrich | T4026 | IB | 1:10,000 |

IB: Immunoblot, IF: Immunofluorescent staining,

**Supplemental Experimental Material and Methods**

**Real time PCR (qPCR)**

The Human Reference Gene Panel plates (Roche Applied Bioscience, Germany, Cat n. 05339545001) containing the 19 candidate genes, 3 positive and 2 negative controls were used for reverse transcription of cDNAs. The panel of pretested qPCR assays for human refeference genes, consists of ready to use qPCR assays, supplied in a LightCycler Instrument 480 Multiwell Plate. All these assays have been designed using the same design algorithm and have been extensively tested ([http://www.roche-applied-science.com](http://www.roche-applied-science.com/)).

The qPCR was carried out as previously detailed (38,39,59,60), using 50 ng of cDNA, according to the manufacturers protocol ([http://www.roche-applied-science.com](http://www.roche-applied-science.com/)). Briefly, qPCR reactions were run in 96-well reaction plates, and qPCR was performed with 50 ng cDNA in a 20 μl reaction mix containing 15 μl Probe Master Master Mix (2×) (Roche Applied Bioscience, Germany). Thermal cycling conditions included 1 cycle at 95 °C for 10 min, followed by 45 cycles of amplification at 95 °C for 10 s, 60 °C for 30 s, 72 °C for 1 s, 40° C for 30 s. The threshold cycle (C_t_) was calculated by the instrument software automatically (threshold value at 0.2). Raw fluorescent data (normalized reporter values, Rn values) were also exported.

For target gene studies, qPCR was performed with Step One Detection System (Applied Biosystems) according to manufacturers protocol, using the TaqMan Universal PCR master mix (# 4304437). For each sample we designed a duplicate assay. For validation studies, *RPLP0*, *PPIA* or *S18* were used as reference genes, and *RPLP0* was selected as the reference gene of *GSK-3β* .

# Characterization of the Analysis Programs

To compare gene expression stability and rank, geNorm (54), NormFinder (55), and BestKeeper (56) algorithms were applied.

**geNorm Analysis.** This program requires the transformation of Ct values by the 2^-ΔΔCt^ method, using the lowest Ct as a calibrator. geNorm computes all possible average pairwise variations between the candidate gene transformed Ct values and provides a measure of the expression stability (M) of each gene. An M-value below 1.5 identifies stable reference genes. geNorm then performs stepwise exclusion of the gene with the highest M-value (least stably expressed gene) and recalculates M-values for the remaining genes. This process permits to rank candidate genes based on their stability of expression, and computes the optimal number of reference genes required for accurate normalization by calculating Vn/n+1 pairwise variations between consecutively ranked normalization factors NFn and NFn+1, where n and n+1 are the number of genes considered, and NFi are the geometric means of the best candidate reference gene transformed Ct values. A pairwise variation of 0.15 is suggested as a cut-off value below which the inclusion of an additional reference gene is not required for reliable normalization (54).

# Normfinder analysis

To calculate the stability value with NormFinder program, for each gene the average Ct value of each duplicate reaction was converted to relative quantity data as described for geNorm (55). According to the analysis, the lowest stability value is top ranked.

# BestKeeper analysis

The BestKeeper program (56) is an Excel-based software tool like geNorm and NormFinder. The program determines the coefficient of correlation analysis for all pairs of candidate reference genes (#8 genes), and calculates the % coefficient of variation (CV) and standard deviation (SD) for each candidate gene’s crossing point (CP) value (the raw quantification cycle value; Cq) (56).

**Cresyl Violet staining**

To count the absolute number of cells present in the granule cell layer in each section, adjacent sections were stained with Cresyl Violet acetate (0.1 w/v) to stain the Nissl substance (38,39). Briefly, hippocampal sections were put into Cresyl Violet acetate solution for 10-15 min. The sections were then rinsed 2 x 5 min in ddH2O and dehydrated in ascending grades of alcohol to xylene and were then rehydrated in decreasing grades of alcohol to xylene and cover-slipped using DPX mounting media. the DG was imaged first at 2X and then at 20-40X. The images were stitched using Adobe Photoshop and analyzed in ImageJ. Calibration slides were taken at each magnification so accurate measurements could be taken. The number of positively stained Cresyl Violet cells were manually counted at 20X magnification in ImageJ. Results (Mean ± SD) are expressed as percentages of cresyl violet positive cells counted in controls (CT = 100).

**Preparation of protein extract**

For hippocampal protein extract preparation, the frozen hippocampi were sonicated in 10X volume/weight of radioimmunoprecipitation assay (RIPA) buffer containing 50 mM Tris-HCl, pH 7.4, 1 mM EDTA, 150 mM NaCl, 0.5% sodium deoxycholate, 1% NP- 40, phosphatase inhibitors (1 mM sodium vanadate and 1 mM sodium fluoride), and protease inhibitors (Proteases Inhibitors Cocktail P8340, Sigma-Aldrich, 10 ml/ml, and 1 mM PMSF). The samples were then centrifuged at 20,000 g for 20 min at 4°C, and the supernatants were recuperated and kept stored at -80°C. For hippocampal neuronal cultures, cells were harvested in ice-cold RIPA buffer containing phosphatase and protease inhibitors (as above). The lysate was then centrifuged at 20,000 g for 20 min at 4°C, and the supernatant was collected, analyzed for protein content and stored at -80°C until immunoblotting analyses.

**Author contributions**

Conceived and designed the experiments: FL,JD-O,RB,FC,BM

Performed the experiments: FL,JD-O,CT, AP,RG,NT,SC,MFS,GC,

Analyzed the data: FL,JD-O,CT,AP,RG,SC,MFS,GS,BM

Contributed reagents/materials/analytical tools: FL,JD-O,CT,AP,RG,FC,BM

Wrote the manuscript: FL,JD-O,FC,RB,BM

**Acknowledgements and Funding.** The authors wish to thank the Italian Ministry of Health (Ricerca Corrente 2013-2015 to BM), the Italian Ministry of Research and University (MIUR, to B.M.) and the OASI (IRCCS) Institution Troina (EN) Italy. Part of this research was further funded by a Canadian Institutes of Health Research grant to FC who is also a recipient of a National Researcher career award from the Fonds de recherche du Québec en santé (FRQS) providing salary support and operating funds. JDO was supported by a post-doctoral fellowship from FRQS. The authors would like to thank the Cambridge Brain Bank for the post mortem tissue which is supported by a grant to the NIHR Cambridge Biomedical Research Centre.
