# Supplementary material for: GSK-3β-induced Tau pathology drives hippocampal neuronal cell death in Huntington's disease: involvement of astrocyte–neuron interactions
Source: Cell Death Dis. 2016 Apr 28;7(4):e2206–. doi: 10.1038/cddis.2016.104 (PMC4855649; doi:10.1038/cddis.2016.104)
Supplement: Supplementary Figures [file cddis2016104x2.pdf]

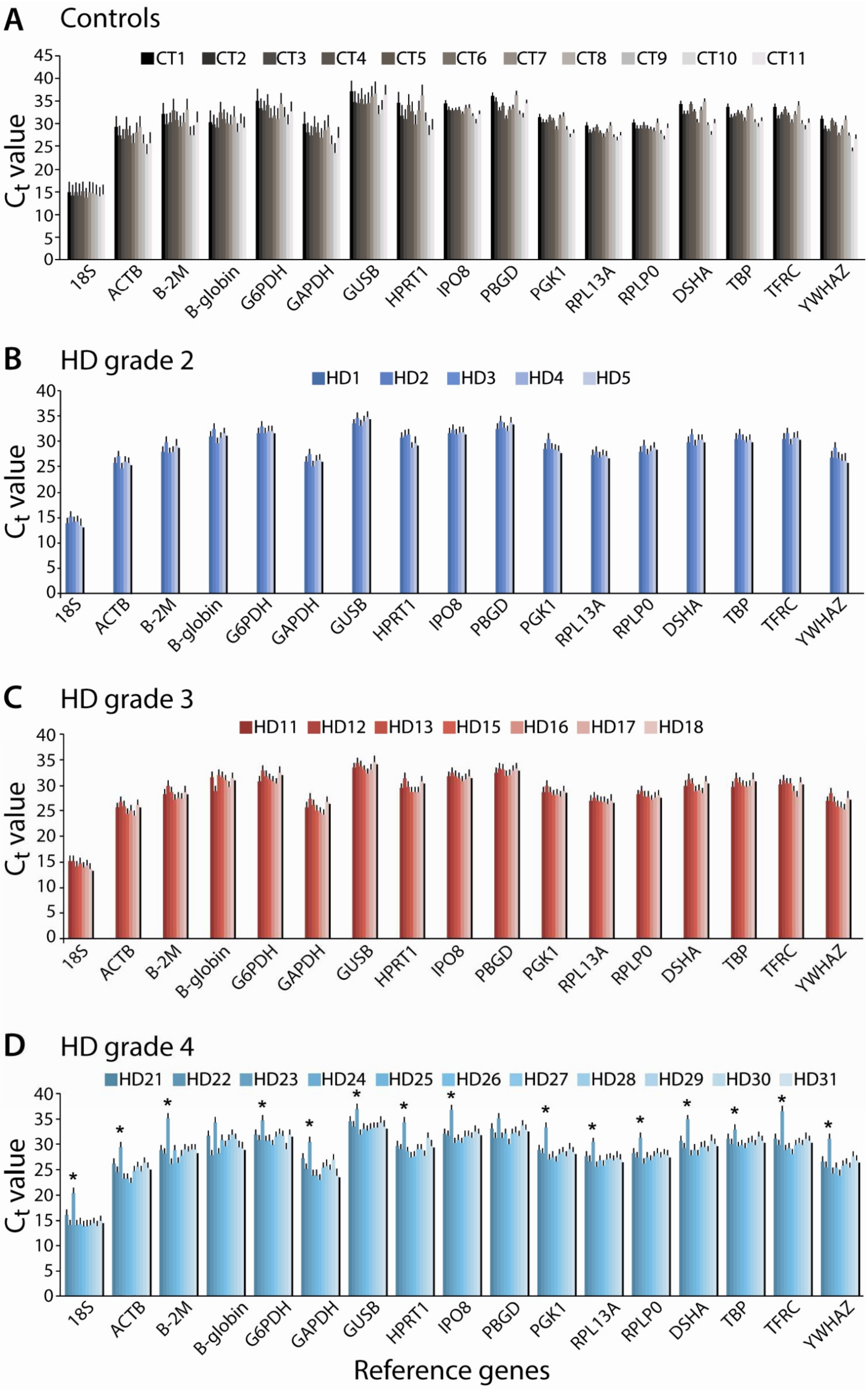

Supplemental Figure 1

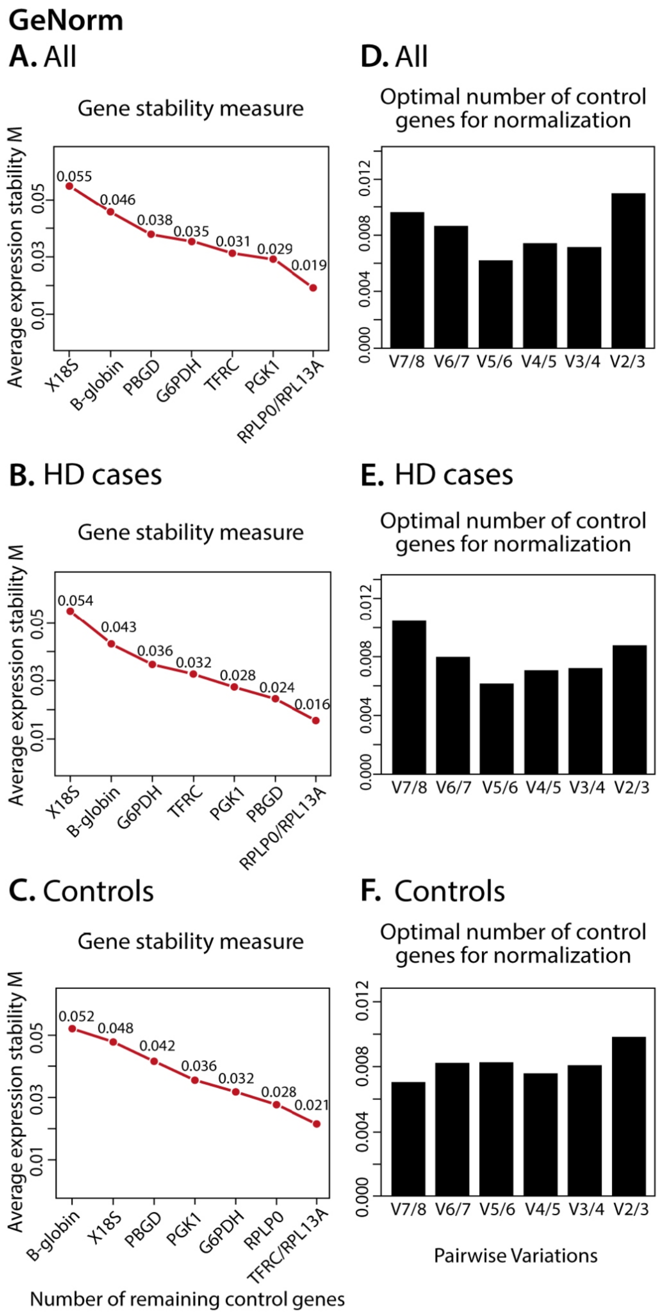

Supplemental Figure 2

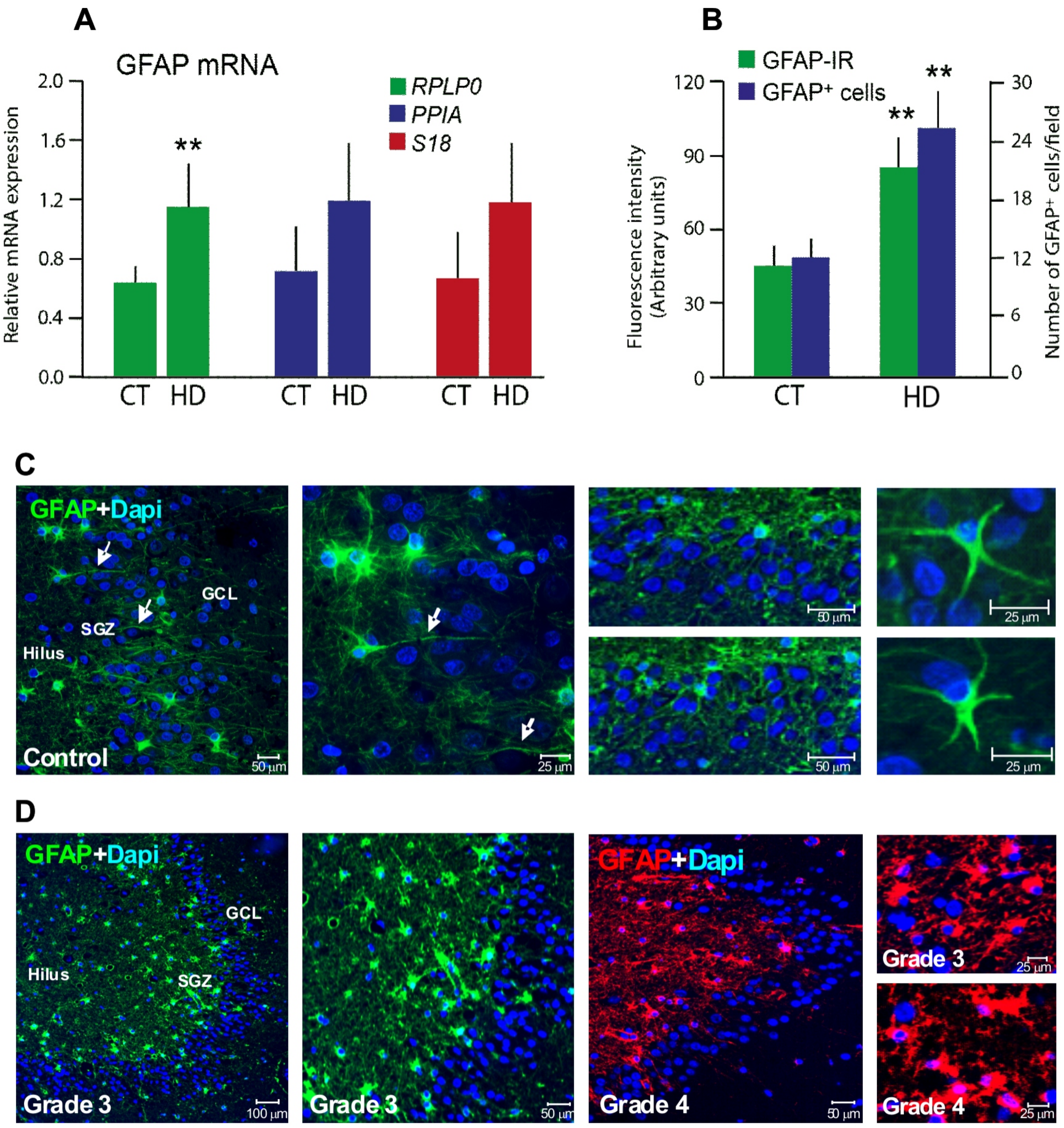

Supplemental Figure 3

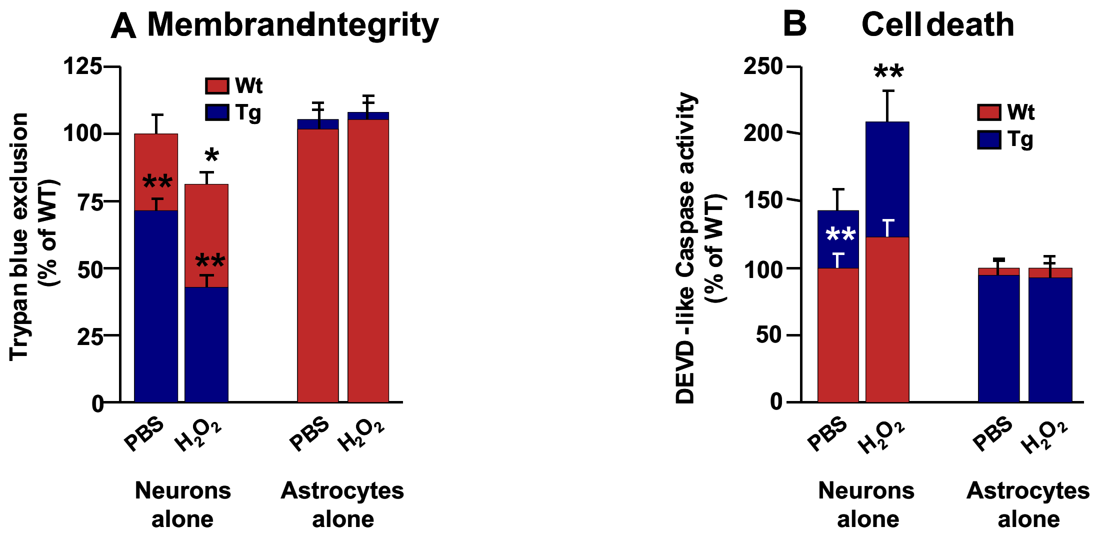

Supplemental Figure 4

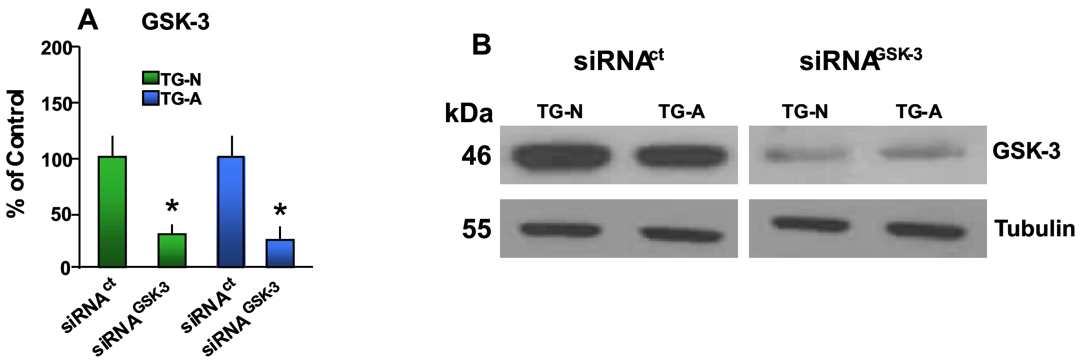

Supplemental Figure 5
